# Meta-analysis of targeted temperature management in animal models of cardiac arrest

**DOI:** 10.1186/s40635-019-0291-9

**Published:** 2020-01-17

**Authors:** Hilmer Olai, Gustav Thornéus, Hannah Watson, Malcolm Macleod, Jonathan Rhodes, Hans Friberg, Niklas Nielsen, Tobias Cronberg, Tomas Deierborg

**Affiliations:** 1 Department of Experimental Medical Science, Experimental Neuroinflammation Laboratory, Lund University, Lund, Sweden; 2 Department of Anaesthesia, Western General Hospital, NHS Lothian, Edinburgh, UK; 3 Department of Critical Care, Western General Hospital, NHS Lothian, Edinburgh, UK; 4 Centre for Clinical Brain Sciences, University of Edinburgh, Edinburgh, UK; 5 Department of Anaesthesia, Critical care and Pain Medicine/NHS Lothian, University of Edinburgh, Edinburgh, UK; 6 Department of Clinical Sciences, Anesthesia & Intensive care, Skåne University Hospital, Lund University, Lund, Sweden; 7 Department of Clinical Sciences Lund, Anesthesia & Intensive care, Helsingborg Hospital, Lund University, Lund, Sweden; 8 Department of Clinical Sciences Lund, Neurology, Skåne University Hospital, Lund University, Lund, Sweden

**Keywords:** Targeted temperature management, Hypothermia, Cardiac arrest, Global ischemia, Animals, Meta-analysis

## Abstract

**Background:**

Targeted temperature management (TTM) of 32 to 34 °C has been the standard treatment for out-of-hospital cardiac arrest since clinical trials in 2002 indicated benefit on survival and neurological outcome. In 2013, a clinical trial showed no difference in outcome between TTM of 33 °C and TTM of 36 °C. In this meta-analysis, we investigate the evidence for TTM in animal models of cardiac arrest.

**Methods:**

We searched PubMed and EMBASE for adult animal studies using TTM as a treatment in different models of cardiac arrest or global brain ischemia which reported neurobehavioural outcome, brain histology or mortality. We used a random effects model to calculate estimates of efficacy and assessed risk of bias using an adapted eight-item version of the Collaborative Approach to Meta-Analysis and Review of Animal Data from Experimental Studies (CAMARADES) quality checklist. We also used a scoring system based on the recommendations of the Stroke Treatment Academic Industry Roundtable (STAIR), to assess the scope of testing in the field. Included studies which investigated a post-ischemic induction of TTM had their treatment regimens characterized with regard to depth, duration and time to treatment and scored against the modified STAIR criteria.

**Results:**

The initial and updated search generated 17809 studies after duplicate removal. One hundred eighty-one studies met the inclusion criteria, including data from 1,787, 6,495 and 2,945 animals for neurobehavioural, histological and mortality outcomes, respectively. TTM was favoured compared to control for all outcomes. TTM was beneficial using short and prolonged cooling, deep and moderate temperature reduction, and early and delayed time to treatment. Median [IQR] study quality was 4 [3 to 6]. Eighteen studies checked seven or more of the eight CAMARADES quality items. There was no clear correlation between study quality and efficacy for any outcome. STAIR analysis identified 102 studies investigating post-ischemic induction of TTM, comprising 147 different treatment regimens of TTM. Only 2 and 8 out of 147 regimens investigated comorbid and gyrencephalic animals, respectively.

**Conclusions:**

TTM is beneficial under most experimental conditions in animal models of cardiac arrest or global brain ischemia. However, research on gyrencephalic species and especially comorbid animals is uncommon and a possible translational gap. Also, low study quality suggests risk of bias within studies. Future animal research should focus on mimicking the clinical scenario and employ similar rigour in trial design to that of modern clinical trials.

## Introduction

Animal research has shown that body temperature may have an effect on the extent of brain damage following global ischemia [1–5]. Hyperthermia is associated with increased damage in animal models [2, 6]. A similar association between hyperthermia and worse neurological outcome in several brain injuries, including cardiac arrest, has been observed in humans [7, 8]. In contrast, hypothermia has been reported to have a protective effect in several animal models [1, 5, 9, 10].

In 2002, two randomized clinical trials (RCT) testing the effects of hypothermia in patients with out-of-hospital cardiac arrest (OHCA) with ventricular fibrillation were published [11, 12]. They showed improved neurological function [11, 12] and increased survival [11] in patients cooled to 32 to 34 °C for 12 to 24 h compared to patients with no temperature control. Hypothermia has since been the recommended treatment in international guidelines [13]. A third RCT published in 2013 further investigated the concept of hypothermia in adults with OHCA, irrespective of initial rhythm. The Targeted Temperature Management (TTM) trial found no benefit from hypothermia at 33 °C compared to controlled temperature at 36 °C for several parameters: survival [14], neurological function [14–16] and release of biomarkers [17, 18]. A similar pediatric study reported no significant increase in survival with good functional outcome at 1 year with hypothermia [19]. In another study, neurological function and mortality was comparable when adults were cooled for 24 and 48 h [20]. In addition, six RCTs investigating prehospital cooling found no benefit of an early induction of hypothermia [21–26]. A recently published RCT, in patients with OHCA and in-hospital cardiac arrest with nonshockable rhythms, compared a controlled temperature of 33 °C to a controlled temperature of 37 °C and found that survival with good neurological outcome was higher with the lower temperature of 33 °C, while overall mortality was similar between the groups [27]. Importantly, in 2015, the European Resuscitation Council and American Heart Association updated their guidelines on post-resuscitation care to include an option for a constant target temperature between 32 °C and 36 °C [28, 29].

The difficulty in translating promising therapies from bench to bedside is hardly new. Among others, the Stroke Treatment Academic Industry Roundtable’s (STAIR) and Collaborative Approach to Meta-Analysis and Review of Animal Data from Experimental Studies (CAMARADES) have developed recommendations for study design and quality to foster translation of new drugs [30, 31].

### Objectives

To our knowledge, there is no pre-clinical meta-analysis studying the efficacy of TTM in experimental models of cardiac arrest or global brain ischemia. Our aims of this meta-analysis are to (i) assess the efficacy of TTM in animal models of cardiac arrest or global brain ischemia (reported as neurobehavioural outcome, brain histology and/or mortality), (ii) assess the experimental conditions modifying efficacy, (iii) assess the quality of individual studies, and (iv) assess the scope of testing in this field. With the results of this study, we hope to elucidate possible knowledge gaps in the translation from animal studies to clinical trials.

## Methods

The methods of this study followed those outlined in a pre-specified study protocol [32]. Departures from the protocol are described in the “Limitations” section and Additional file 1.

### Search strategy and eligibility

EMBASE and PubMed were searched with no restriction to time of publication or language. The search contained three blocks: (i) synonyms to cardiac arrest or ischemia (broad search) AND (ii) synonyms to hypothermia or temperature manipulation (in title and/or abstract) AND (iii) synonyms to brain or its relevant structures (broad search), see Additional file 1 for details. The first database search was performed 29 August 2015, and it was updated 23 September 2016. A hand search of references from the included studies was performed as well as a hand search of five selected reviews [33–37].

Studies were included if they were controlled, induced global brain ischemia in an adult (sexually mature) non-human animal (mammal), used TTM as a treatment, and assessed neurobehavioural outcome, mortality or a histological assessment of neuronal death/injury in brain tissues. Studies were excluded if they used historical controls, induced TTM pharmacologically, used cooling/heating only to prevent spontaneous temperature change without a corresponding control group, where data could not be used for meta-analysis (e.g. no information on group size or variance), which treated animals with therapies adjuvant to TTM, treated newborn animals, and investigated deep hypothermic circulatory arrest or cardiopulmonary bypass to enable cardiothoracic surgery. Further details are given in the supplement.

### Data extraction and outcome assessment

Studies were entered into EndNote X7 and exported to CAMARADES Data Manager (Microsoft Access), which was used to screen abstracts and review studies in full text. Two independent reviewers screened each abstract. HO, GT and HW performed this screening. Studies which two reviewers agreed on to exclude were excluded, and the remaining studies were reviewed in full text independently by HO and GT. Questions regarding eligibility were solved by consulting TD, NN or JR. The references from included studies and selected reviews were entered into Microsoft Excel. References already screened and studies not mentioning a synonym to ischemia or a synonym to TTM in the title were removed by HO. Abstracts of remaining studies were screened by HO. Full-text review were performed independently by HO and GT.

Data extraction were performed independently by HO and GT using a data extraction template (see Additional file 1). Disagreements were solved by discussion, and if not, then with the assistance of TD. Software from CAMARADES-NC3Rs Preclinical Systematic Review & Meta-analysis Facility (SyRF), available at app.syrf.org.uk, was used for data extraction and synthesis.

Our primary outcome was neurobehaviour. Brain histology and mortality were secondary outcomes. If more than one histological or neurobehavioural outcome were reported from the same cohort of animals, we summarized these using fixed-effects meta-analysis to provide a summary estimate of each outcome. When a single control group served multiple treatment groups, the number of animals in the control group was divided by the number of treatment groups which it served and recorded as the actual number [38]. If outcome was measured serially, only the final measure was extracted. Ordinal data was analyzed as continuous variables for the purpose of the meta-analysis. If data were presented in graphical form only, computerized ruler software was used to measure graphs (FlexRuler version 2.3, DropFrame, 2015).

Individual studies were checked using an adapted version of the CAMARADES quality checklist [30], consisting of (i) publication in a peer-reviewed journal, (ii) randomization to treatment or control, (iii) blinded induction of ischemia (i.e. concealment of treatment group allocation at time of induction of ischemia), (iv) blinded assessment of outcome, (v) statement of inclusion and exclusion of animals from the study, (vi) sample size calculation, (vii) statement of compliance with regulatory requirements, and (viii) statement regarding possible conflicts of interest. One study could check a maximum of eight items, indicating a potentially lower risk of bias compared to a study checking zero items.

We used modified STAIR criteria to assess the scope of testing in the subset of studies with post-ischemic induction of TTM. It is similar to the one constructed by O’Collins et al. to evaluate neuroprotective drugs in models of stroke [31, 33]. Our criteria consisted of eight items: (i) laboratory setting—regimen tested in two or more laboratories, (ii) animal species—regimen tested in lissencephalic (rodents and rabbits) and gyrencephalic species, (iii) health of animals—regimen tested in comorbid animals (hyperglycemic, aged, hypertensive), (iv) sex of animals—regimen tested in male and female animals, (v) outcome measures—regimen evaluated with both histology and neurobehaviour, (vi) long-term effect—regimen evaluated long-term outcome (≥ 4 weeks with either histology, mortality or neurobehaviour), (vii) route of delivery—regimen tested with two or more methods of TTM (e.g. intravascular cooling, surface cooling), and (viii) injury model—regimen tested in two or more models of global brain ischemia where at least one model of global ischemia is accomplished by induced cardiac arrest. To visualize the scope of animal testing of clinical strategies of TTM, we constructed three 3 × 3-matrixes (< 2, 2 to 6, > 6 h time to treatment post-ischemia), each with three pre-specified durations and temperature intervals. Each regimen was checked against the STAIR criteria and contributed to the score of a specific combination of time to treatment, duration and depth of TTM. A specific combination could score from zero to eight, with eight being the highest possible, indicating a more extensive scope of testing (for details, see Additional file 1).

### Statistical analysis

For neurobehavioural and histological outcome, we used standardized mean difference (SMD), where a value above zero is interpreted as favouring the intervention. For mortality, we used logarithmic odds ratio, where a value above zero is interpreted as favouring the intervention. We used a random effects model as we expected great inter-study heterogeneity, and *I* square (*I*^2^) to measure heterogeneity for the global estimate of each outcome. This analysis was performed in the Shiny app for SyRF [39]. Publication bias was measured with funnel plot, Egger regression and trim and fill.

Stratified subgroup analysis was pre-specified and divided into two domains, study design and quality, with 16 and 5 items, respectively (see Additional file 1 for details). To account for multiple comparisons, we set a significance level at *p* < 0.01 for the quality domain and *p* < 0.0031 for the design domain. The significance of differences between *n* groups was measured by partitioning heterogeneity and by using Chi-square test, *n* − 1 degrees of freedom.

## Results

### Study selection

**Fig. 1:**
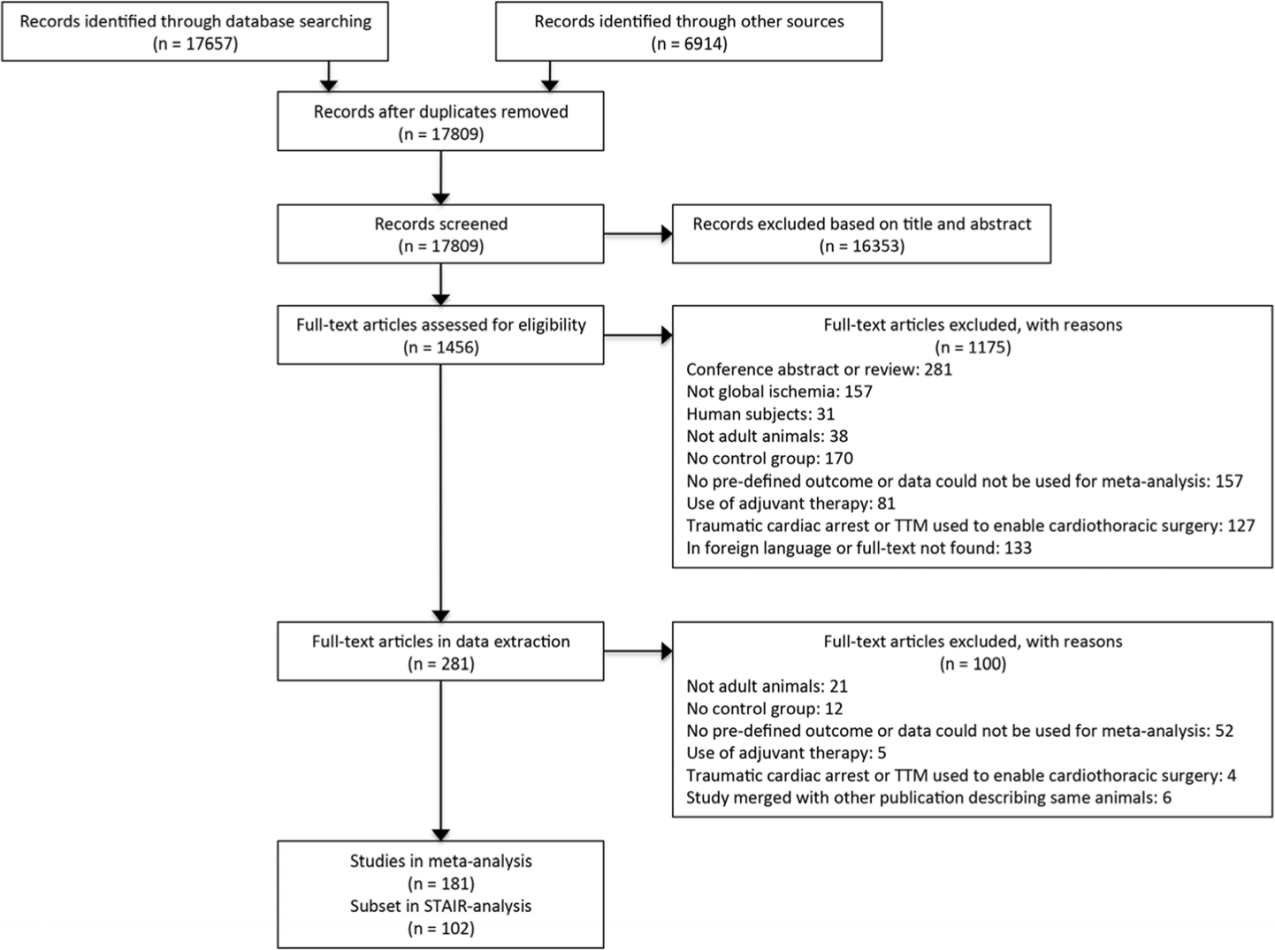
PRISMA flow diagram

There were 7048 and 10609 studies generated from PubMed and EMBASE, respectively. The hand search of included articles and selected reviews generated 5939 and 975 studies, respectively. After exclusion of 16353 and 1275 studies during abstract screening and full-text review, respectively, a total of 181 studies were included in the meta-analysis (173 from database search, 8 from hand search), 102 of which investigated post-ischemic induction of TTM and were included in the STAIR analysis (Fig. 1, see Additional file 1 for the full list of references). Altogether, the studies described 779 comparisons of treatment and control (123, 481 and 175 comparisons of neurobehaviour, histology and mortality in 1787, 6495 and 2945 animals, respectively).

We consulted a veterinarian to confirm if swine reasonably could be considered sexually mature on the basis of the reported weights; many studies were excluded as a result.

### Study characteristics and quality

Neurobehavioural outcomes extracted were mostly overall performance categories (OPC) and neurobehavioural deficit scores (NDS) but also tests such as open field (motor/anxious behaviour) and Morris water maze (cognition/memory). The hippocampal CA1-area was the most common histological area evaluated, comprising 59% of all histological comparisons. Histological evaluation differed in use of staining method (Hematoxylin & Eosin, TUNEL, Cresyl violet and NeuN being common stains). Induced normothermia was by far the most common way of managing temperature in the control group. Median [IQR] times to outcome evaluation in days were 4 [3 to 14], 7 [4 to 7] and 5 [3 to 7] for neurobehavioural, histological and mortality outcomes, respectively. Median times to treatment in minutes in relation to time of recirculation or return of spontaneous circulation (ROSC) were 1 [− 4.4 to 8.4], 1 [− 20 to 30] and 1 [− 2.4 to 30] for neurobehavioural, histological and mortality outcomes, respectively. Median depths of TTM in degrees Celsius were 33 [32 to 34], 33 [31.3 to 33.6] and 33 [32 to 33.5] for neurobehavioural, histological and mortality outcomes, respectively. Lastly, median durations of TTM in hours were 3 [1.1 to 11], 1.9 [0.8 to 5.3] and 4.1 [1.2 to 23] for neurobehavioural, histological and mortality outcomes, respectively.

The median number of items checked in the CAMARADES quality checklist was 4 [3 to 6]. Only three studies met all eight items, and 15 studies met seven items.

### Efficacy and publication bias

TTM was favoured compared to control for all three outcomes. Global estimates of efficacy were 0.93 [95% CI 0.79 to 1.08] SMD for neurobehaviour and 1.52 [95% CI 1.42 to 1.63] SMD for histology. The logarithmic odds ratio was 1.03 [95% CI 0.84 to 1.21] for mortality. There was substantial heterogeneity for neurobehavioural (*I*^2^ = 47%) and histological (*I*^2^ = 62%) outcomes but not for mortality (*I*^2^ = 0.7%).

**Fig. 2:**
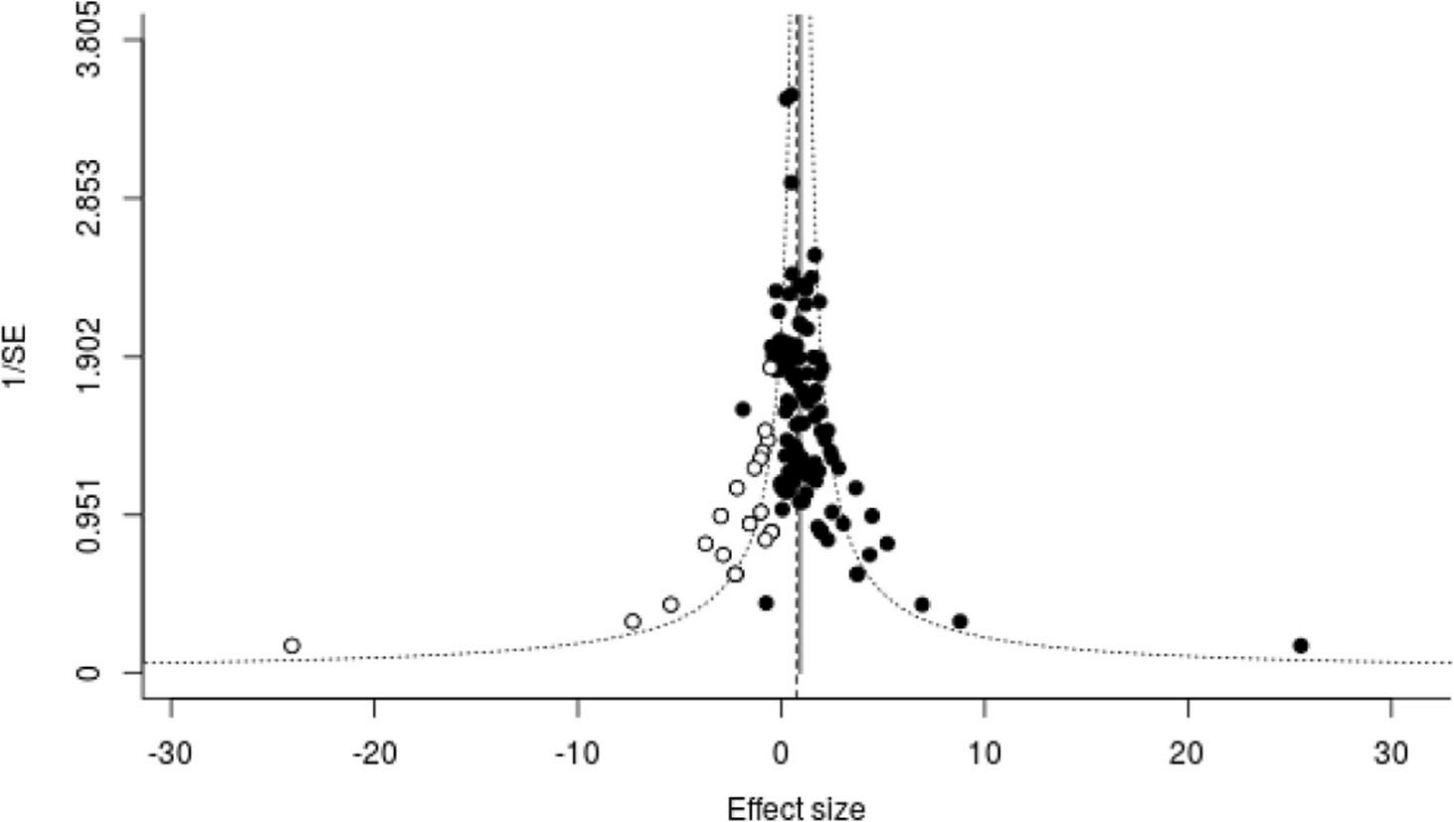
Funnel plot and Trim and Fill analysis of neurobehavioural outcome. Open circles are missing studies according to Trim and Fill-analysis. Estimate of efficacy with missing studies included: 0.76 SMD [95% CI 0.59 to 0.93]. Efficacy without missing studies: 0.93 SMD [95% CI 0.79 to 1.08]. Standardized mean difference = SMD. Standard error = SE. Confidence interval = CI

Trim and fill-analysis and Egger regression was consistent with the presence of publication bias for neurobehavioural and histological outcomes; however, estimates of efficacy still significantly favoured TTM compared to control in Trim and Fill-analysis (Fig. 2 and Additional file 1). No evidence of publication bias was found for the mortality outcome.

### Aspects of study quality modifying efficacy

**Fig. 3:**
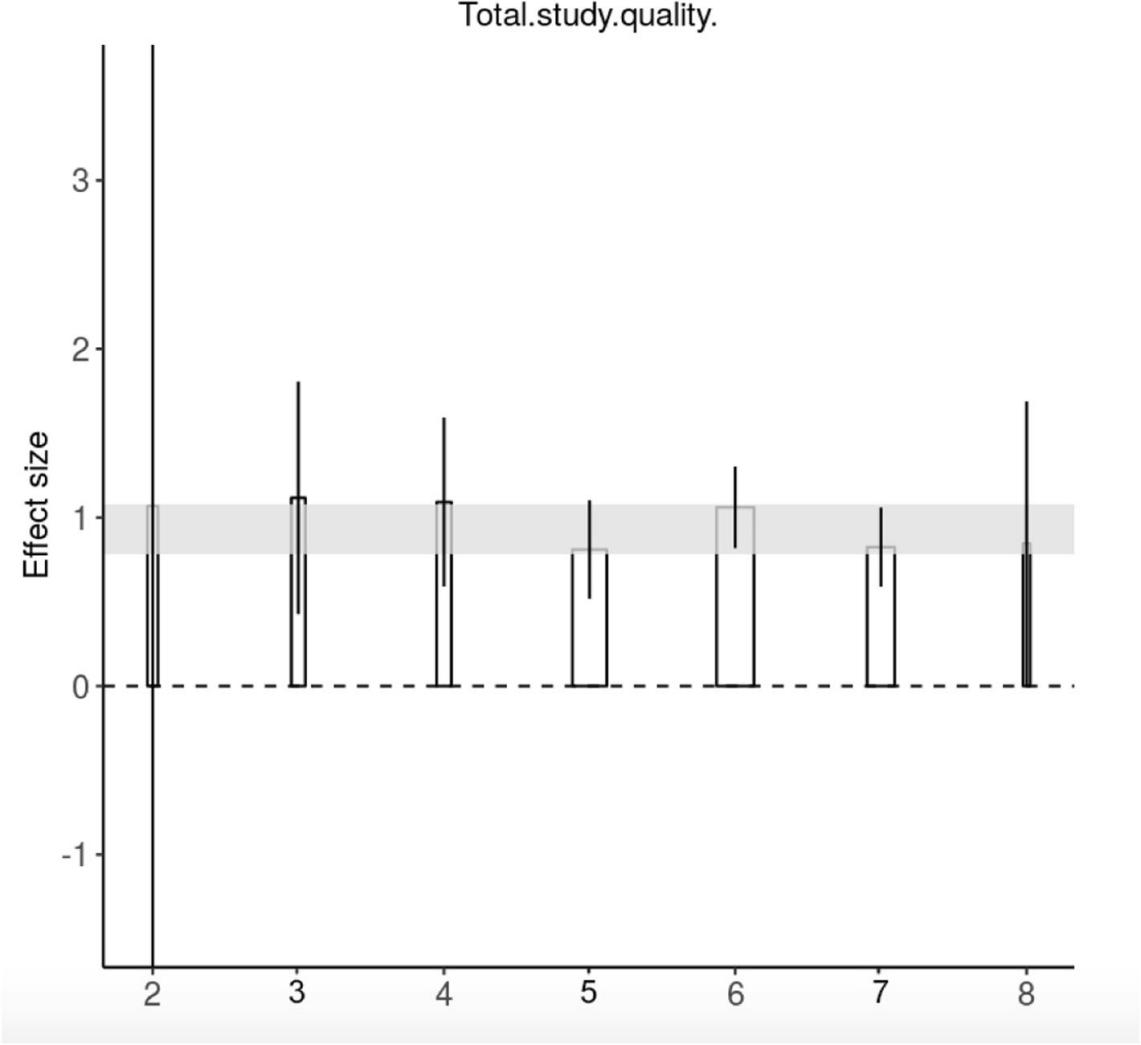
Number of study quality checklist items scored and point estimates of SMD with 95% confidence intervals (CI) for neurobehavioural outcome. Low numerical checklist score on *x* axis indicates higher risk of bias. Shaded area is the 95% CI of the global estimate. Thickness of bar reflects number of contributing comparisons. Score ranging from a minimum of 0 to a maximum of 8. SMD = standardized mean difference

**Fig. 4:**
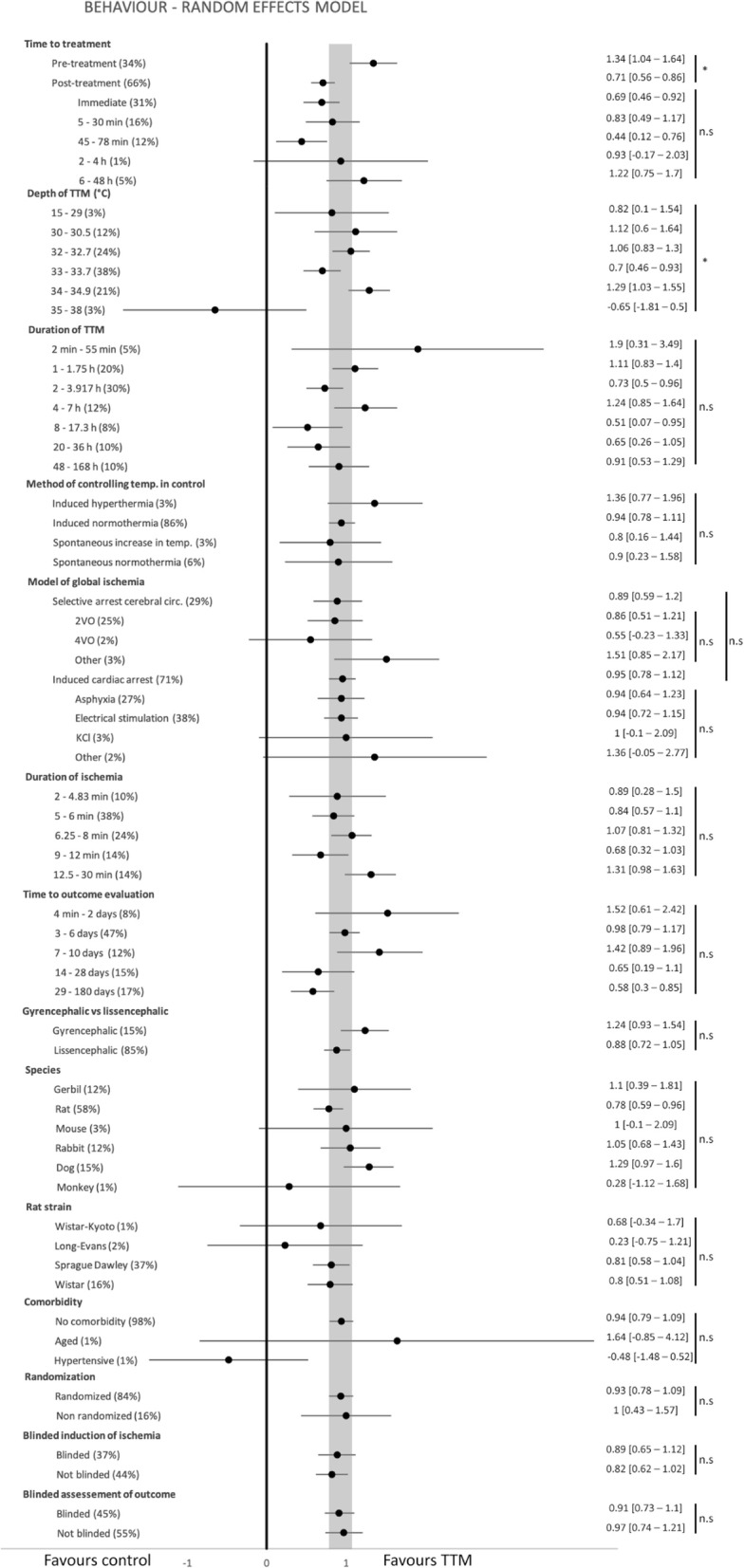
Forest plot for neurobehavioural outcome (SMD) and 95% confidence intervals (CI) for select subgroups. Shaded area is the 95% CI of the global estimate. Percentage in parentheses is meta-analytic weight. “Unknown” categories are omitted. Right column brackets are 95% CI’s. *p < 0.0031, #*p* < 0.01 and n.s (not significant) denote between-group differences for the groups covered by the vertical line. Corresponding forest plots for histological and mortality outcomes are in supplement. SMD = standardized mean difference

There was no clear correlation between effect size and total study quality (Fig. 3 and Additional file 1). Reporting of randomization was not associated with differences in efficacy for neurobehavioural and mortality outcomes, but efficacy was lower in randomized studies reporting histological outcomes (Fig. 4 and Additional file 1). Similarly, reporting of the blinded assessment of outcome was not associated with differences in neurobehavioural outcomes, but efficacy was lower in blinded studies reporting histological outcomes.

### Aspects of study design modifying efficacy

TTM was superior to control under most experimental conditions. The control procedure was not significantly favoured for any subgroup (see forest plot in Fig. 4 and Additional file 1). There were few clear findings relating to dose or aspects of study design which were observed across all three outcomes; one more robust finding was that induction of TTM before recirculation or ROSC was more effective than when induction occurred after ischemia, although this too showed efficacy across all outcomes (Fig. 4 and Additional file 1). A longer post-ischemic delay of TTM was not clearly less effective than early post-ischemic induction. For the histological outcome, maximal efficacy was seen at around 31 °C, but this was not seen for neurobehaviour and mortality. However, across all outcomes, target temperatures closer to normothermia tended to perform less well. A prolonged duration of TTM appeared to increase efficacy for histological outcomes, but this was not seen for the other two outcomes. Since surface cooling was used in most studies, a comparison between cooling methods was considered less meaningful. TTM was beneficial for all durations of ischemia, and no clear difference of efficacy was seen between shorter and longer durations of ischemia. Outcome evaluation at 4 weeks or later did not reveal any coherent trend for efficacy across outcomes; TTM was more beneficial in histological outcome and less so in neurobehavioural. TTM was beneficial in both gyrencephalic (mostly dogs) and lissencephalic (rodents and rabbits) species. Comorbid testing was rarely reported, comprising 2%, 3% and 2% of comparisons for neurobehavioural, histological and mortality outcomes, respectively, without clear evidence for the efficacy of TTM in these animals.

### STAIR analysis

One hundred two studies with post-ischemic induction of TTM described 147 different regimens of TTM with respect to post-ischemic time to treatment, duration and depth. Most (86%) regimens of TTM were administered within 2 h of ischemia, and most frequently (49%) to a depth between 29 and 34 °C and for a duration shorter than 8 h (Table 1). Forty percent of regimens used a model of induced cardiac arrest rather than selective arrest of cerebral circulation. Animals with comorbidities and gyrencephalic animals were used in 1% and 5% of regimens, respectively. TTM was induced by surface cooling alone in 82% of regimens, and 19% of regimens assessed outcome at 4 weeks or later.

**Table 1:**
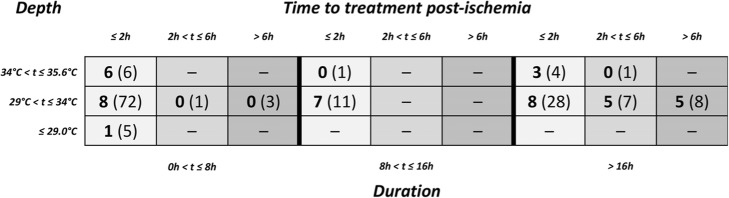
STAIR analysis of 102 studies with post-ischemic induction of TTM, which described 147 different regimens. The score is presented as *x* (*y*), where *x* is the number of items checked and (*y*) is the number of treatment regimens contributing to that score, where 8 is the highest possible score. Empty boxes were not tested

## Discussion

The quantitative results from this meta-analysis suggest that TTM, as a treatment in animal models of cardiac arrest, is beneficial with regard to clinically relevant outcomes such as neurobehaviour and mortality but also brain histology under most experimental conditions. Substantial between-study heterogeneity and generally low to moderate study quality require these results to be interpreted with considerable caution. The descriptive part of our study reveals that an extensive range of experiments have been performed but also suggests some overlooked aspects which might be relevant for translation to the clinical setting.

### Study quality

We expected that measures to minimize bias might reduce the efficacy of TTM. This was only evident for randomization procedures and blinded outcome assessment of the histological outcome, similar to the results in another preclinical meta-analysis for stroke models [40]. We were somewhat surprised that no convincing decrease of efficacy was observed with increasing total study quality. However, a “meta”-meta-analysis investigating the effect on outcome by total study quality showed no difference between high- and low-quality studies [41], and it has been argued that total study quality is to be regarded as a checklist rather than a quantitative measure [41, 42].

### Study design

No single, optimal regimen of TTM was apparent. Rather, regardless of timing, duration and depth, TTM was superior to control.

Studies on rodents with selective arrest of cerebral circulation have suggested that post-ischemic induction of TTM has outcomes comparable to normothermic controls, while pre- and intra-ischemic TTM are greatly beneficial [1, 2, 43–45]. This contrasts with results from studies, claiming that longer durations of TTM are protective even when administered post-ischemia and also at longer survival times [3, 46, 47]. Subsequent studies of rodents using induced cardiac arrest models have showed that longer durations of TTM are needed when administered post-ischemia [48–51]. Studies on dogs with cardiac arrest have showed benefits of TTM when induced at the start of CPR, but when delayed only for 15 min post-ischemia, benefits were abolished [4, 5, 52, 53]. Our results support the superiority of TTM induced before recirculation or ROSC but also show that post-ischemic induction seems beneficial.

Studies claiming a benefit of post-ischemic TTM have estimated a therapeutic window up to 4 to 12 h from recirculation or ROSC with decreasing efficacy with increasing delay [3, 46–48, 54]. Our results support this fairly wide therapeutic window, allowing a delay of more than 6 h post-ischemia for neurobehavioural and histological outcomes and up to 4 h for mortality.

Most studies comparing short-term and long-term outcome showed decreasing efficacy with time, some with significant protection compared to control [46, 47, 55] while others did not show long-term protection of TTM [43, 56]. Our results are not entirely consistent, with lower and higher estimates of efficacy of TTM seen for neurobehavioural and histological outcomes, respectively, when evaluation is performed after 4 weeks. TTM is not significantly different from control when there is a late evaluation of mortality.

When several depths of TTM were tested, a lower temperature around 30 to 33 °C was sometimes favoured compared to “milder” TTM around 34 to 35 °C [2, 57–59], but just as often there was no significant difference between lower and “milder” TTM [60–62]. In the preclinical meta-analysis on TTM as treatment of ischemic stroke, efficacy was greater at temperatures below 31 °C [40]. We found no such sweet spot of depth except around 31 to 32 °C for the histological outcome.

As for duration of TTM, studies addressing this issue tended to favour longer TTM typically around 24 h [63–65]. Our results showed no such trends except for the histological outcome where durations beyond 8 h showed increasing efficacy.

Since our meta-analysis could not confirm any intuitive dose-response relationships of different aspects of TTM across outcomes—which we anticipated given the findings in studies investigating dose-response—we cannot conclude on an optimal regimen of TTM.

### STAIR analysis

Case studies of accidental hypothermia where patients survived prolonged no-flow states when cooled to very low temperatures before cardiac arrest occurred, and the use of deep hypothermic circulatory arrest in cardiothoracic surgery also enabling long periods of no-flow otherwise impossible in normothermic subjects, demonstrate beyond doubt that pre-ischemic induction of TTM has neuroprotective effects in humans [66–68]. A clinically feasible regimen of TTM in victims of cardiac arrest is usually post-ischemic, or possibly intra-ischemic. The testing of post-ischemic TTM in animal studies is extensive and matches the regimens used in the three larger clinical trials [11, 12, 14]. The STAIR analysis also revealed a low use of comorbid animals (1%) and gyrencephalic species (5%). In the clinical trials, patients were old and had comorbidities, with average ages of 64 [14], 59 [11] and 67 years [12]. Ischemic heart disease and diabetes were present in around 30% and 10% in the TTM-trial and the HACA study. The few studies that evaluated animals with comorbidities did not consistently favour TTM [69–72]. Outcome was evaluated at 6 months in both the TTM-trial and the HACA study, a time point rare in animal studies; only 14 of 481 histological comparisons were made at 6 months or later and no comparisons of neurobehaviour and mortality were made at this time.

### Possible translational gaps

Many studies had been conducted to explore injury mechanisms, i.e. they were not primarily designed to evaluate survival with good neurological function but focused on cellular and molecular mechanisms responsible for neuronal injury. In these studies, the models are often focused on a limited brain area (i.e. hippocampus CA1) and titrated to produce a minimal loss of animals and mortality is not regularly reported. We suggest that the mechanistic studies are less relevant to the translation of TTM to the clinic, compared to studies with a stated intent of clinical translation. It is difficult to discern to what extent this contribution of mechanistic studies might skew the results of this meta-analysis, but we consider it a possible translational gap.

While clinically relevant aspects of experimental design have been evaluated separately, we did not find a single study using comorbid animals in a cardiac arrest model with a post-ischemic delay of TTM and functional outcome assessment at around 6 months, mimicking the clinical scenario. Also, studies of gyrencephalic species (dogs and swine) evaluated outcome relatively early at around 3 to 4 days, likely due to costs of animal handling. A problem for future research could be that to simulate the high rate of mortality of real-life cardiac arrest in larger, comorbid animal models would not be considered ethical.

The STAIR analysis shows extensive testing, in particular of regimens with a time to treatment of less than 2 h post-ischemia. The majority of these regimens are closer to immediate induction than to 2 h. In the clinical setting, TTM is commonly induced around 2 h post-ischemia [11, 20, 73]. Induction of TTM beyond 2 h is not equally well tested, and here, comorbid and gyrencephalic testing is lacking. The predominant use of very early induction of TTM in animal studies, rarely achieved in the clinical setting, might also be a translational gap. However, quantitative subgroup analysis did show benefit of TTM induced beyond 2 h.

## Limitations

Most papers are not written with a future meta-analysis in mind. Therefore, to characterize how TTM was delivered was sometimes difficult. This might have led to errors in interpretation and data extraction. We believe the research field would benefit from adhering to the Utstein-guidelines for laboratory cardiopulmonary resuscitation research [74] and the development of a standardized format for describing TTM. A standardized format of TTM could include calculations of the area under the curve to estimate the “dose,” which would make comparisons of different regimens easier.

Induced normothermia was the most common method of managing the control animals. Studies differed in how long they controlled and registered temperature. Late temperature fluctuations after the end of registration are possible confounders in many experiments [75] and subsequently in this meta-analysis. There is also an ongoing discussion whether a controlled temperature of 36 °C is to be regarded as a very mild form of hypothermia rather than normothermia, which if true, would change the interpretation of a majority of animal research [76].

We were fairly liberal in our interpretation of processes of randomization (46%) and blinding of outcomes (59%), only requiring a simple statement, which might have led to overestimation. Demanding more detail would have drastically decreased the number of studies checking these items.

Contrary to the neurobehavioral and histological outcome, *I*^2^ was very low for the mortality outcome, implying low study heterogeneity. This may reflect a “tuning” of study designs to minimize the need for animals to be euthanized prior to the planned end of the experiment, both for animal welfare reasons and to minimize attrition (animals not able to contribute data for analysis). The impression during review, however, was that studies had substantial heterogeneity in the reported aspects of study design, so the lack of statistical heterogeneity is somewhat surprising.

Although this study analyzes a large sample of studies, it does not provide a complete view. Since the database search was updated, new studies have surely been published. We were not able to retrieve all studies identified by the search; moreover, we did not translate studies written in a non-English language, which might introduce bias to our results. If data from a study were incomplete, we decided against contacting the authors for clarification. Therefore, the results reflect the data possible to extract solely from the studies as published. Other protocol deviations can be found in the supplement.

Since the publication of our protocol, new findings based on data from focal ischemia experiments have been published [77], suggesting that normalized mean difference as effect size and meta-regression produces higher statistical power than standardized mean difference and partitioning heterogeneity. This might be applicable to global ischemia experiments, possibly limiting our results.

Lastly, the fairly wide inclusion criteria including many different species as well as different models of global brain ischemia catches the scope of testing while on the other hand limits the quantitative analysis since the experimental settings are different and not always comparable. Also, some aspects of study design, such as method of CPR, were not recorded. A few studies used cardiopulmonary bypass to resuscitate animals [4, 52, 53, 60], a factor that could influence our results.

## Conclusions

The large body of quantitative data from this meta-analysis shows that TTM as a treatment of cardiac arrest is favoured under almost all experimental conditions and for all outcomes. However, a majority of animals were healthy rodents and many studies were of low study quality increasing the risk of bias. Also, the large amount of study heterogeneity weaken the quantitative results overall. Clinical trials have used the treatment strategies suggested by animal experimentation, but it is possible that animal experimentation has not simulated the clinical scenario well enough.

## Supplementary information

**Additional file 1.** Details of inclusion and exclusion criteria; deviations from the study protocol; bar plots for histological and mortality outcomes, total study quality; trim and fill-analysis for histological and mortality outcome; comprehensive forest plots for neurobehavioural, and histological and mortality outcomes.

## Data Availability

The raw data can be provided upon request.
