# Supplementary material for: Meta-analysis of targeted temperature management in animal models of cardiac arrest
Source: Intensive Care Med Exp. 2020 Jan 17;8:3. doi: 10.1186/s40635-019-0291-9 (PMC6969098; doi:10.1186/s40635-019-0291-9)
Supplement: Supplementary file 1 — Additional file 1. Details of inclusion and exclusion criteria; deviations from the study protocol; bar plots for histological and mortality outcomes, total study quality; trim and fill-analysis for histological and mortality outcome; comprehensive forest plots for neurobehavioural, and histological and mortality outcomes. [file 40635_2019_291_MOESM1_ESM.docx]

**Supplement**

**Details of inclusion and exclusion criteria**

Conference abstracts and reviews were not included.

Accepted controls were: induced normothermia, induced hyperthermia (to mimic post-ischemic fever), control group spontaneously remained at normothermia after ischemia, control group spontaneously increased its temperature after ischemia and lastly control group with unknown management/temperature verification. Control groups had to be evaluated at the same time as the treatment groups. We did not record the specific equipment used to maintain temperature in the control group or exact temperature, but rather if temperature was actively regulated and if it was considered by authors to be normothermia or hyperthermia.

If no age or weight was stated, animals were assumed adult unless the study clearly modeled neonatal pathology. A veterinarian was consulted if there was any uncertainty about the age; sexual maturity was used as a sign of adulthood.

An experienced animal researcher (TD) evaluated the validity of staining protocols for determining neuronal cell death/injury – blinded to the outcomes. Validity was assessed based on the choice of stain(s), and the choice of structure(s) evaluated. Protocols evaluating non-specific cell distress or glial activity were excluded.

Outcome only presented as the median and interquartile range were not included since the meta-analysis demanded the mean and standard deviation or standard error of the mean.

**Deviations from the study protocol**

If data were incomplete, the protocol stated to endeavor to contact authors. After data extraction was completed a majority of the studies had one or more data pieces missing or unknown. Thus, to contact all authors and receive a response was not feasible with regard to the time frame of this project. Therefore, the results in this analysis reflect the data that were possible to extract solely from the studies as published.

During the process of abstract screening it became clear that the protocol was not clear on whether to include models of traumatic cardiac arrest. The preclinical models of traumatic cardiac arrest investigating TTM differ from the non-traumatic ones, particularly with respect to the use of suspended animation and exsanguination. Since the purpose of this meta-analysis was to investigate the preclinical evidence for the use of TTM in non-traumatic models of cardiac arrest, we chose to exclude traumatic models of cardiac arrest.

All included studies are written in English. The protocol did not state an exclusion of studies in a language other than English. However, due to limited funding we were not able to translate these studies. A total of 133 articles were deemed as possibly relevant based on information in title or abstract, 8 of which were written in English and could not be accessed in full-text. The remaining 125 had an English title or abstract but the full-text written in another language (Chinese, Japanese and Russian being most common), 104 which could not be accessed in full-text.

**Bar plots for histological and mortality outcomes; Total study quality**

Figure S1.


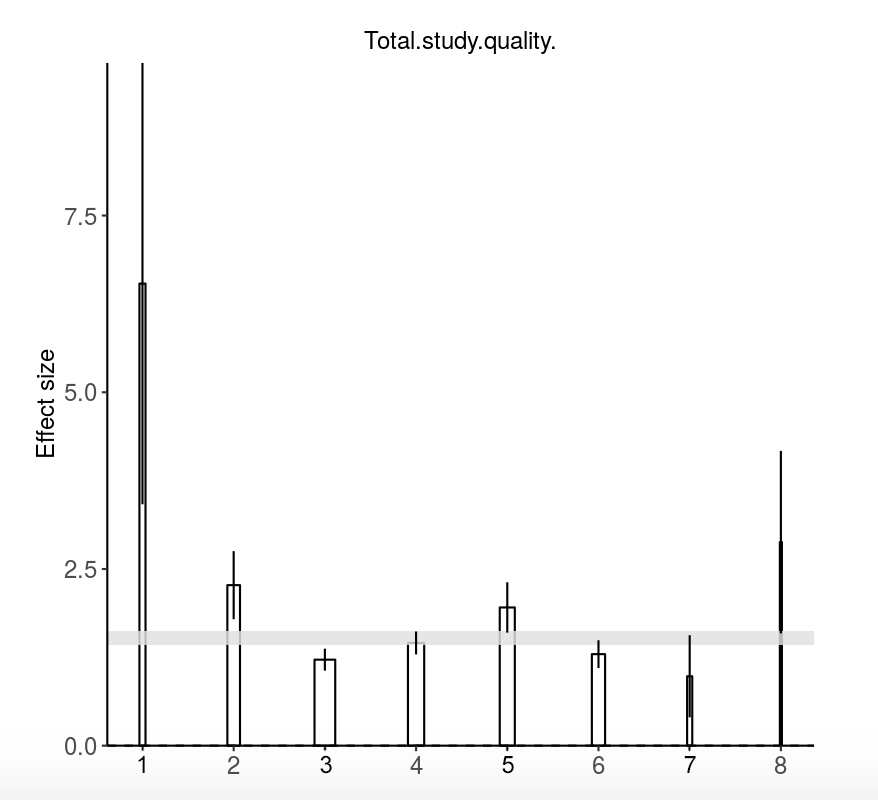


Legend S1: Number of study quality checklist items scored and point estimates of SMD with 95% confidence intervals (CI) for histological outcome. Low numerical checklist score on x-axis indicates higher risk of bias. Shaded area is the 95% CI of the global estimate. Thickness of bar reflects number of contributing comparisons. Score ranging from a minimum of 0 to a maximum of 8. SMD = Standardized mean difference.

Figure S2.


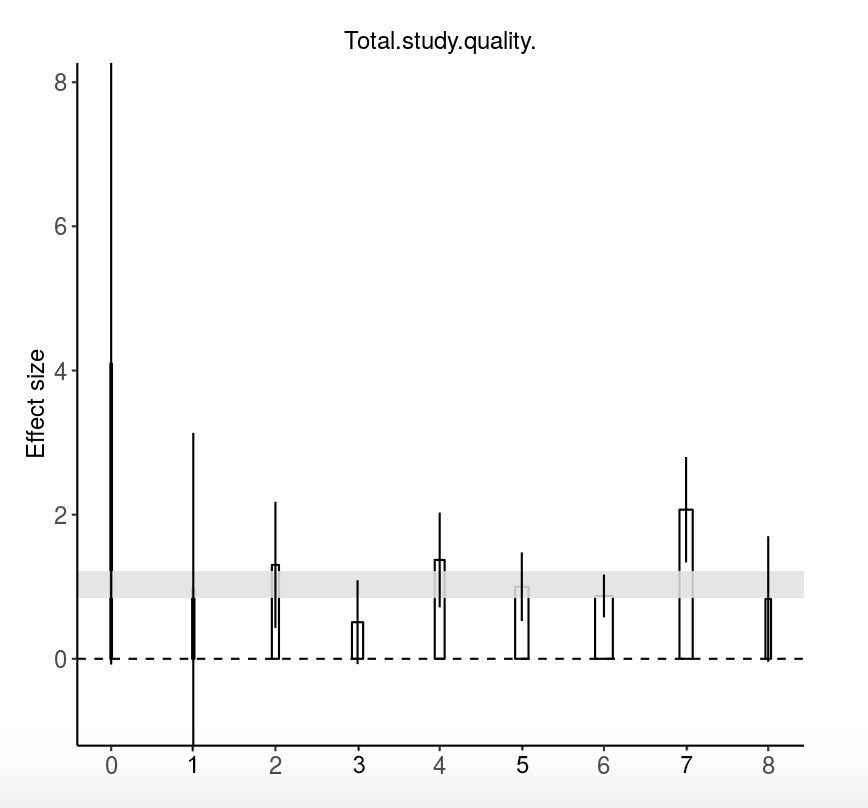


Legend S2: Number of study quality checklist items scored and point estimates of logarithmic odds ratio with 95% confidence intervals (CI) for mortality outcome. Low numerical checklist score on x-axis indicates higher risk of bias. Shaded area is the 95% CI of the global estimate. Thickness of bar reflects number of contributing comparisons. Score ranging from a minimum of 0 to a maximum of 8.

**Trim and fill-analysis for histological and mortality outcome**

Figure S3.


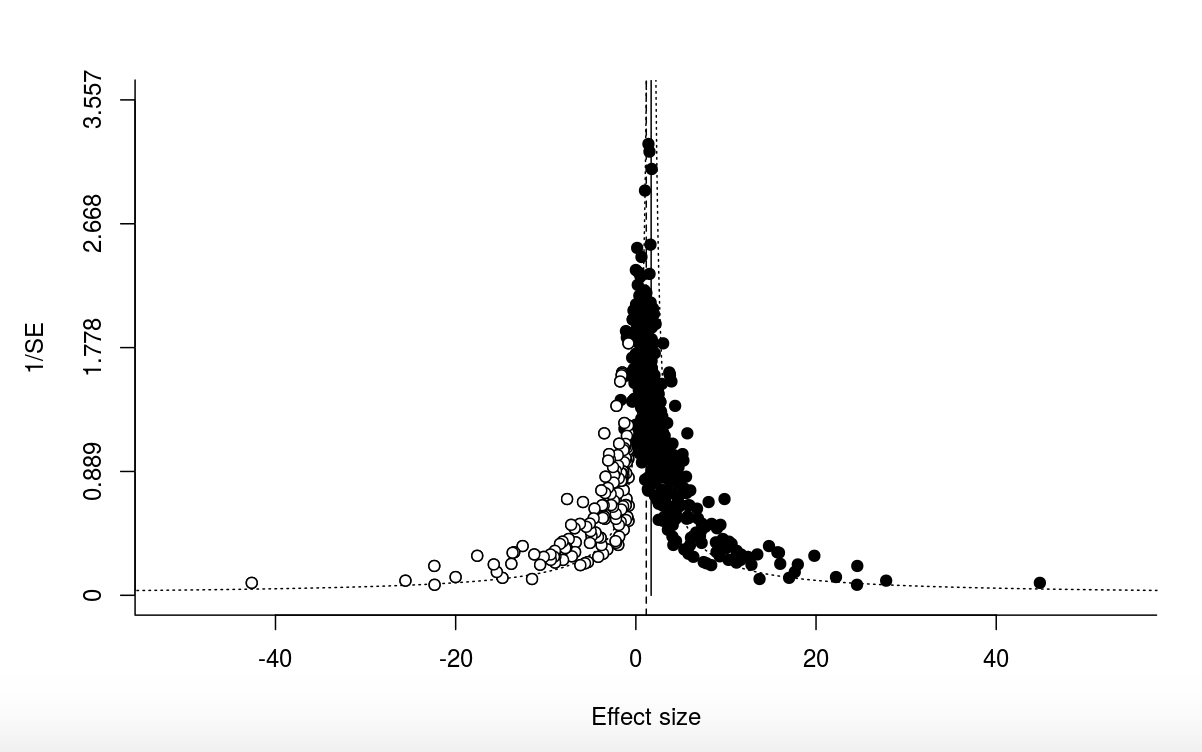


Legend S3: Funnel plot and Trim and Fill-analysis of histological outcome. Open circles are missing studies according to Trim and Fill-analysis. Estimate of efficacy with missing studies included: 1.16 SMD [95% CI 0.97 to 1.34]. Efficacy without missing studies: 1.52 SMD [95% CI 1.42 to 1.63]. Standard error = SE. Confidence interval = CI.

Figure S4.


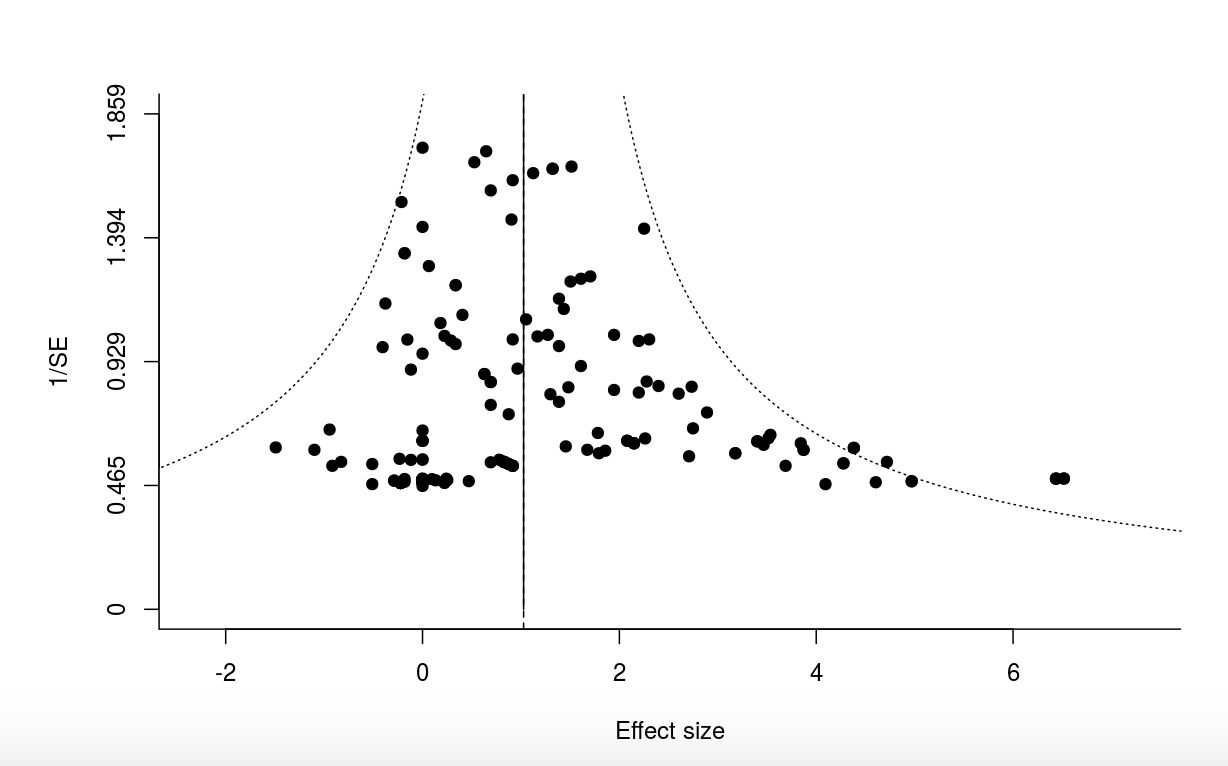


Legend S4: Legend 2: Funnel plot and Trim and Fill-analysis of mortality outcome. Open circles are missing studies according to Trim and Fill-analysis. Estimate of efficacy with missing studies included: 1.03 logarithmic odds ratio [95% CI 0.84 to 1.21]. Efficacy without missing studies: 1.03 [95% CI 0.84 to 1.21]. Standard error = SE. Confidence interval = CI.

**Comprehensive forest plots for neurobehavioural, histological and mortality outcomes**

Figure S5.


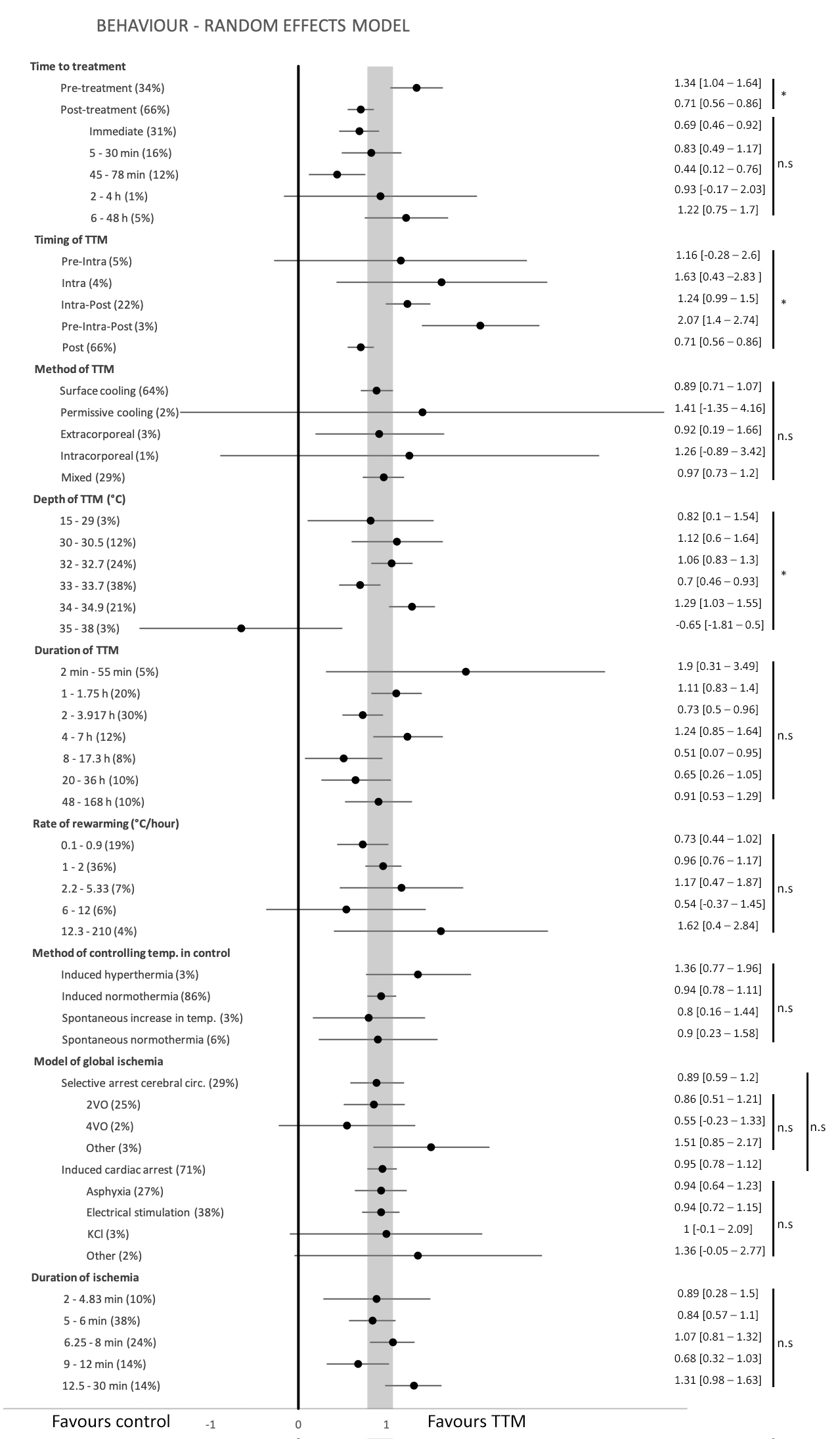


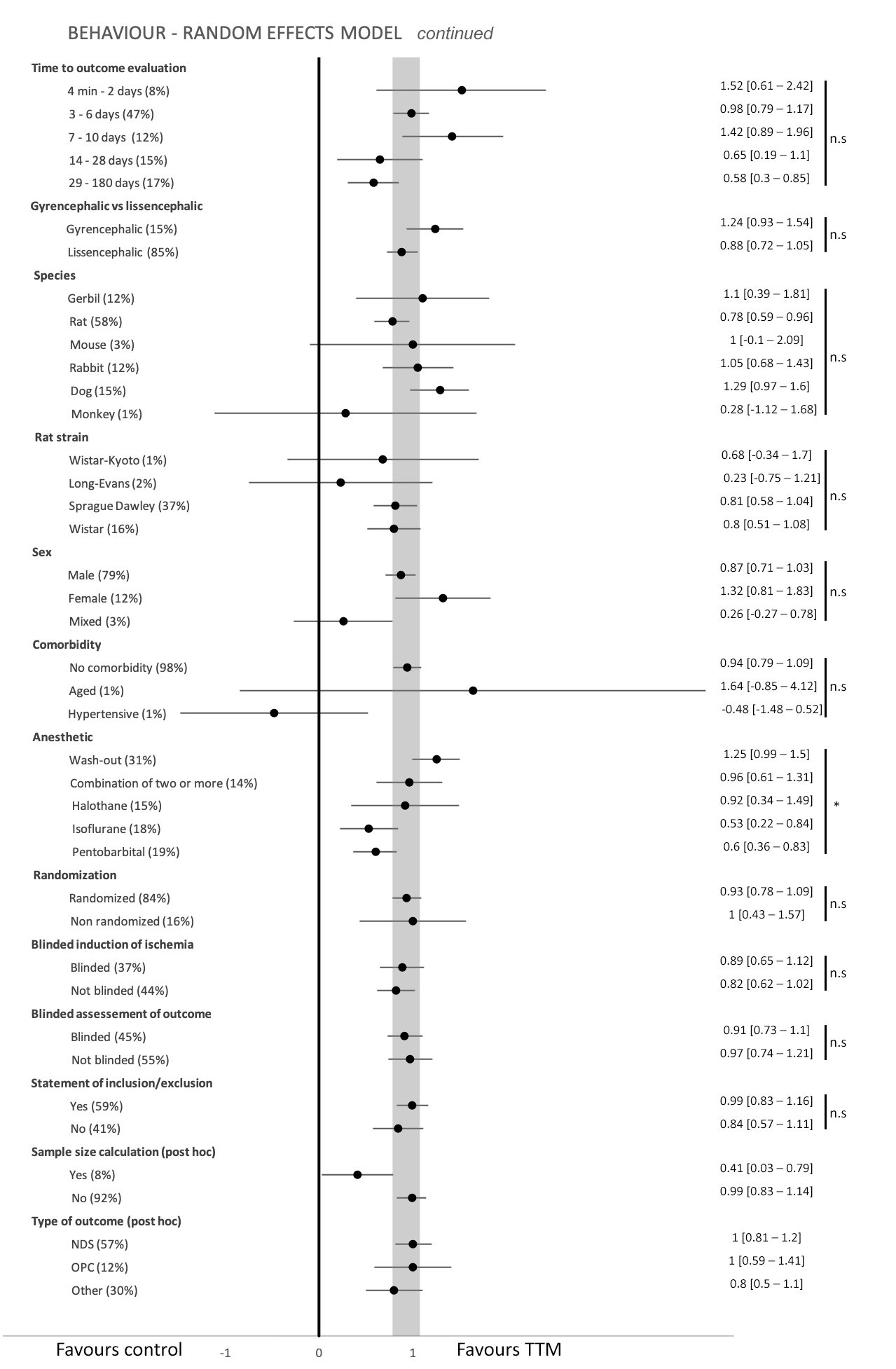


Legend S5: Forest plot for neurobehavioural outcome (SMD) and 95% confidence intervals (CI) for subgroups. Shaded area is the 95% CI of the global estimate. Percentage in parentheses is meta-analytic weight. “Unknown” categories are omitted. Right column brackets are 95% CI’s. * p < 0.0031, # p < 0.01 and n.s (not significant) denotes between-group differences for the groups covered by the vertical line. SMD = Standardized mean difference.

Figure S6.


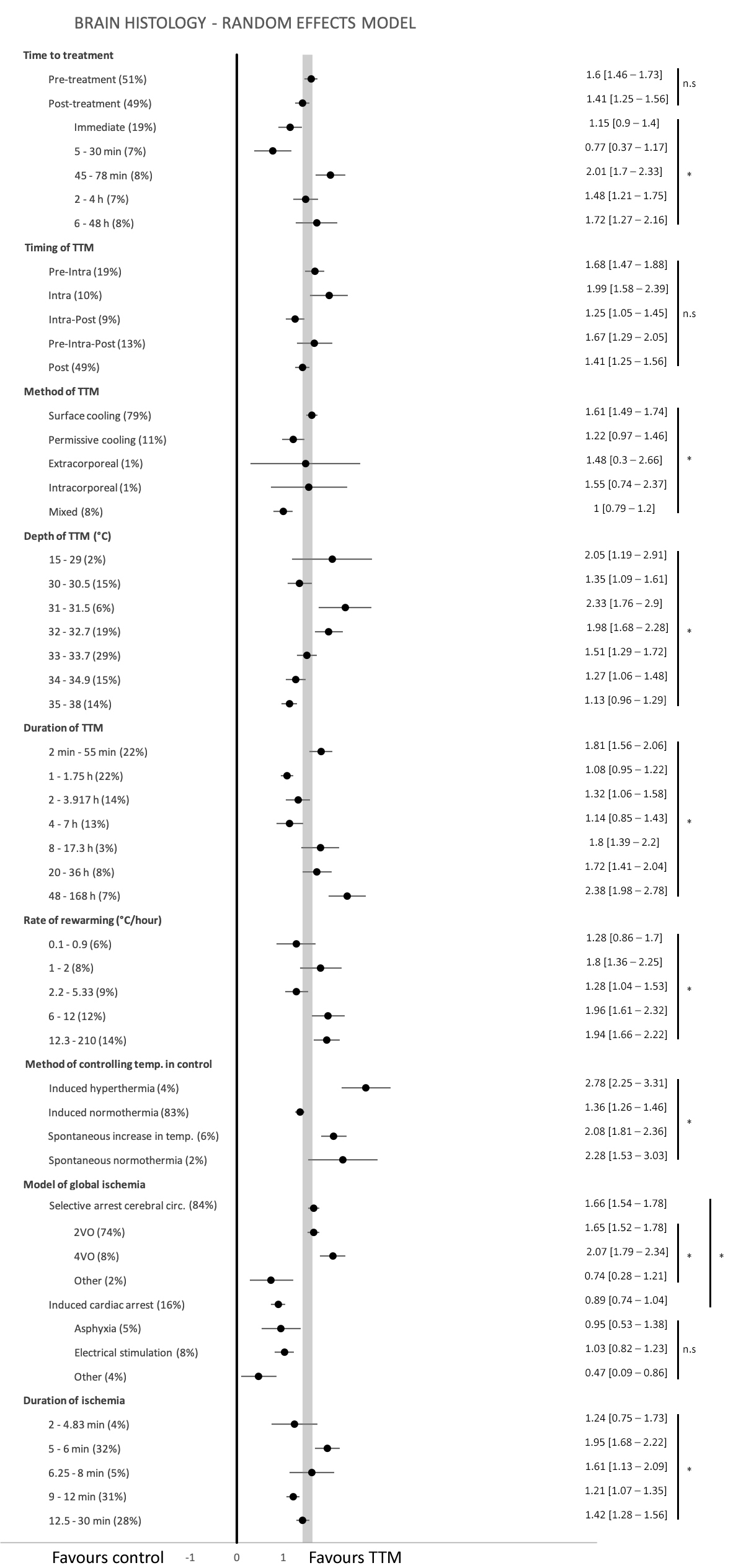


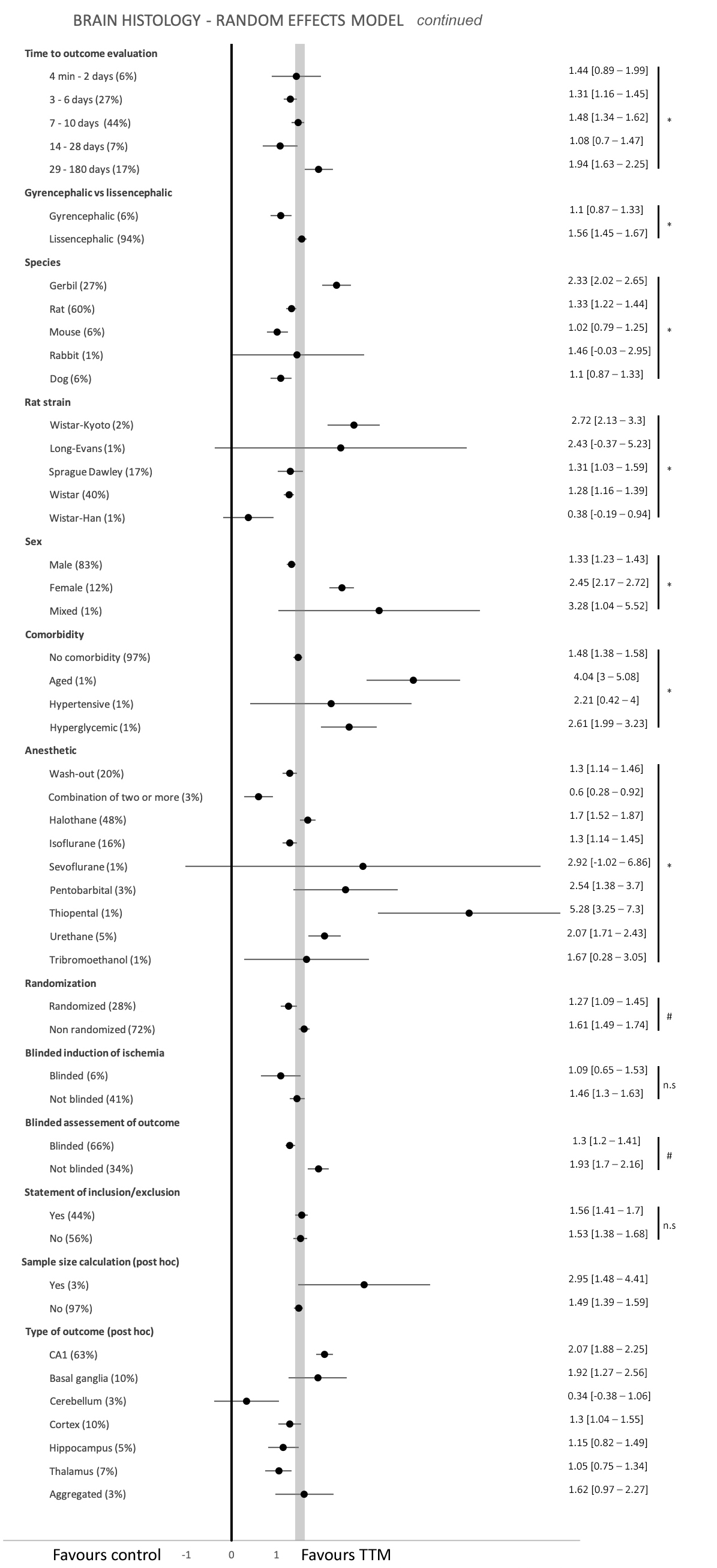


Legend S6: Forest plot for histological outcome (SMD) and 95% confidence intervals (CI) for subgroups. Shaded area is the 95% CI of the global estimate. Percentage in parentheses is meta-analytic weight. “Unknown” categories are omitted. Right column brackets are 95% CI’s. * p < 0.0031, # p < 0.01 and n.s (not significant) denotes between-group differences for the groups covered by the vertical line. SMD = Standardized mean difference.

Figure S7.


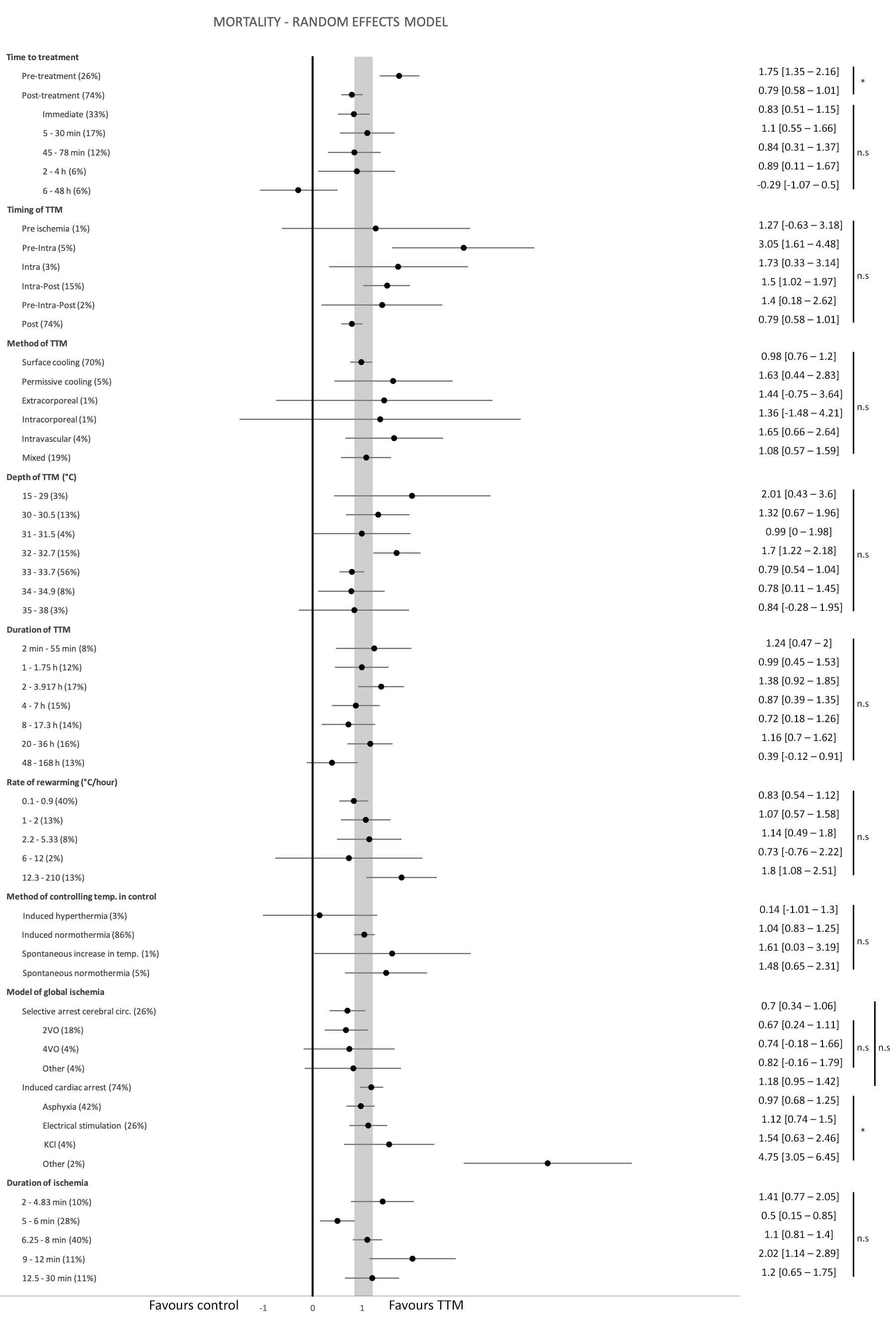


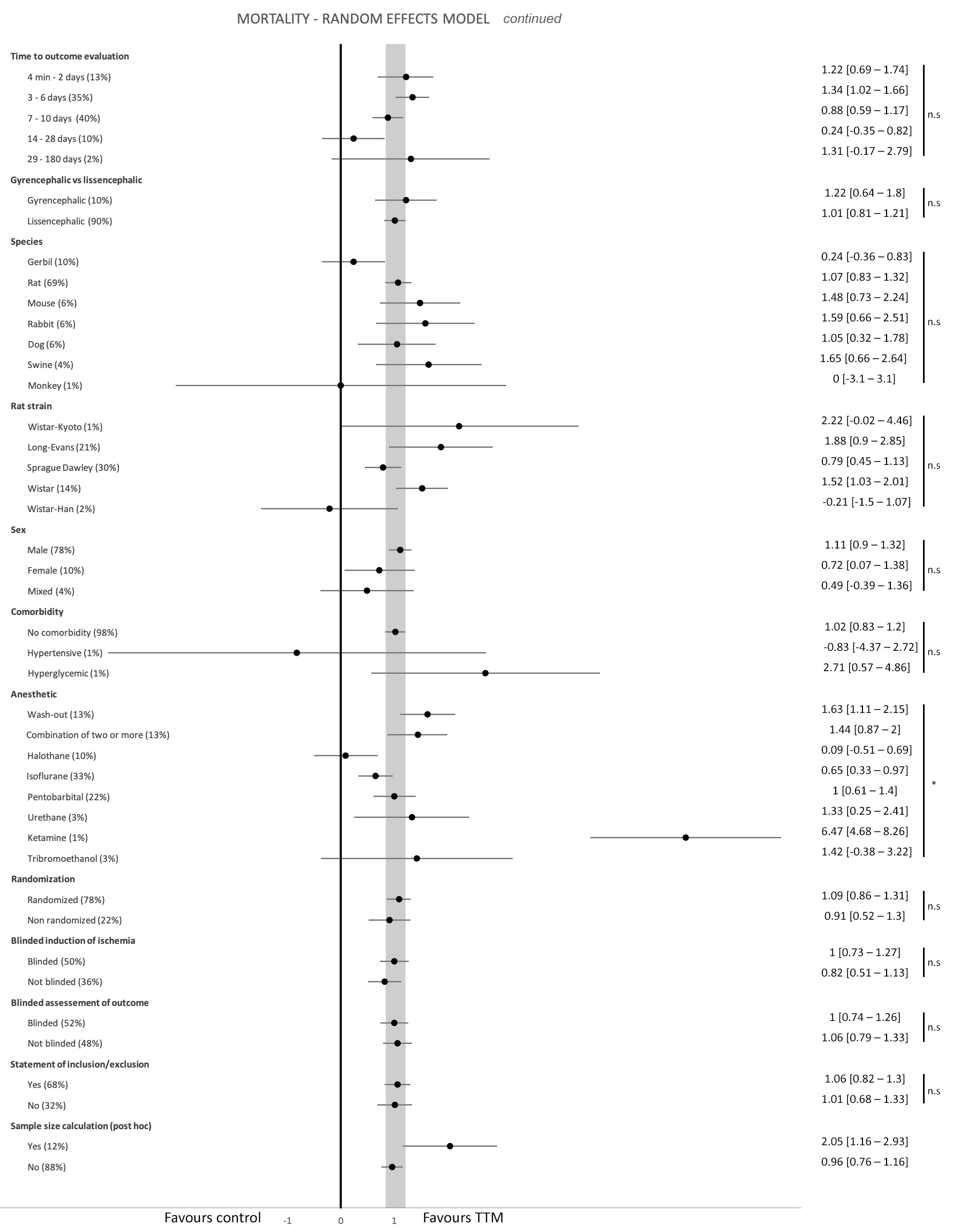


Legend S7: Forest plot for mortality outcome (logarithmic odds ratio) and 95% confidence intervals (CI) for subgroups. Shaded area is the 95% CI of the global estimate. Percentage in parentheses is meta-analytic weight. “Unknown” categories are omitted. Right column brackets are 95% CI’s. * p < 0.0031, # p < 0.01 and n.s (not significant) denotes between-group differences for the groups covered by the vertical line.

**Pre-defined data extraction sheet**

We extracted the following pre-specified data from every study:

General information:

Title;

Authors;

Journal;

Year of publication.

Quantitative data:

Number of treatment and corresponding control groups;

Number of animals in control and treatment groups;

When a single control group served multiple treatment groups, the control group was divided by the number of treatment groups which it served and recorded as the actual number;

Assessments of neurobehaviour, brain histology and mortality – if an outcome was measured serially only the last measurement was extracted;

The mean of the outcome and the standard deviation or the standard error of the mean, including the direction of the outcome, i.e. if greater values were considered worse or not (outcomes reported with median and interquartile ranges were not extracted for the quantitative analysis);

If more than one histological or neurobehavioural outcome were reported for the same cohort of animals (e.g. cell death in different parts of the hippocampus), we summarized these using fixed‐effects meta‐analysis to provide a summary estimate of each outcome;

The time of outcome assessment;

For the mortality outcome the number of dead and alive animals were recorded.

Adapted CAMARADES quality checklist:

Publication in a peer-reviewed journal – based on information from journal homepage or www.ulrichsweb.serialssolutions.com;

Randomization to treatment or control – we accepted a simple statement of randomization and did not require a detailed description of the process;

Blinded induction of ischemia (i.e. concealment of treatment group allocation at time of induction of ischemia) – we accepted a simple statement and if randomization was performed after induction of ischemia, blinded induction was checked;

Blinded assessment of outcome – we accepted a simple statement and if at least one reported outcome assessment was described as blinded, this item was checked;

Statement of inclusion and exclusion of animals from the study – we only accepted explicit statements, e.g. protocol violations leading to exclusion, number of exclusions etc., we did not require a flow chart describing attrition and we did not accept implicit statements;

Sample size calculation – we accepted a simple statement and did not require a detailed description;

Statement of compliance with regulatory requirements;

Statement regarding possible conflicts of interest – to check this item we required a statement of the sources of funding at the very least.

Stratifications:

Design domain:

Timing of TTM – the timing was defined in relation to ischemia, and it could be either pre-, pre & intra-, pre & intra & post-, intra-, intra & post-, post-ischemia or unknown. In models of induced cardiac arrest, where TTM was initiated in the time-window after arrest but before ROSC (i.e. during CPR), we defined it as intra-induction to adhere to current clinical terminology;

Time to treatment (in minutes) – the time to treatment was defined in relation to the time of recirculation (for models of selective arrest of cerebral circulation) or ROSC (for models of induced cardiac arrest). Studies stating immediate induction of TTM post ischemia were assumed to have started at 1 minute post ischemia;

Method of inducing TTM – the method was defined as either surface cooling, intravascular, extracorporeal (ECMO, CPB, dialysis – heat transfer outside the body), intracorporeal (nasopharyngeal, Total Liquid Ventilation, intraperitoneal lavage etc), mixed (when more than one method is used), permissive cooling or unknown;

Depth of TTM (in Celsius) – the target temperature was used if stated, otherwise it was measured in graphs. If temperature was measured at several positions (e.g. rectal, temporalis muscle etc.), the temperature measured closest to brain was used. If a regimen of TTM used several depths in a step-wise fashion, the weighted arithmetic mean was calculated and used;

Duration of TTM (in hours?) – the start of TTM was defined as the start of cooling and the end was defined as the start of rewarming;

Rate of rewarming (in Celsius/hour) – if the rate was stated it was used, otherwise it was measured and calculated from graphs;

Method of rewarming – the method was defined as either surface warming, intravascular, extracorporeal (ECMO, CPB, dialysis – heat transfer outside the body), intracorporeal (nasopharyngeal, Total Liquid Ventilation, intraperitoneal lavage etc), mixed (when more than one method is used), permissive warming or unknown;

Method of controlling temperature in the control group – the method was defined as either induced normothermia (if heating or cooling equipment was used to achieve a normothermic target), induced hyperthermia, control group spontaneously remained at normothermia after ischemia, control group spontaneously increased its temperature after ischemia and lastly control group with unknown management/temperature verification;

Model of global ischemia – the model was defined as either induced cardiac arrest (asphyxia, KCl induced arrest, electrical stimulation leading to ventricular fibrillation etc., requiring chest compressions and/or defibrillations) or selective arrest of the cerebral circulation (bilateral occlusion of carotid arteries, bilateral occlusion of carotid and vertebral arteries etc.). Models of unilateral or permanent occlusion of cerebral arteries were not included. Traumatic models of cardiac arrest, using exsanguination to produce cardiac arrest, were not included;

Duration of ischemia (in minutes) – the stated duration was used. In models of induced cardiac arrest the time from induction of ischemia until the start of resuscitation was defined as duration. In models of asphyxia, the actual time of arrest was used if stated, and if not, the duration of asphyxia was used;

Time to outcome assessment (in days) – the stated time was used. Control and treatment group had to be evaluated at the same time point, e.g. a histological comparison between control and treatment where the controls died or were sacrificed earlier than the treatment, was not included;

Species – the stated species was recorded;

Strain – the stated strain was recorded, and if a transgenic strain was used, we only recorded “transgenic”.

Sex – the stated sex of the animals was recorded, and if both male and female animals were used, “mixed” was recorded;

Comorbid animals – we recorded use of hyperglycemic, aged or hypertensive animals;

Choice of anesthetic – we recorded the stated anesthetic. If more than one anesthetic was used, we recorded “combination of two or more anesthetics”. If an attempt was made to wash out the anesthetic before induction of ischemia we recorded “attempt made to wash-out anesthetic” instead of the specific drug. We did not stratify use of muscle relaxants or other drugs;

Quality domain:

Total study quality – the sum of checked items in the adapted CAMARADES quality checklist, ranging from zero to eight, where eight is the maximum. Note that this is not an ordinal score but rather a number of checklist items scored;

We also stratified four individual items of the study quality checklist – randomization to treatment or control, blinded induction of ischemia, blinded assessment of outcome and statement of inclusion and exclusion of animals from the study. For the stratification of blinded assessment of outcome, we required that the extracted outcome was described as blinded;

Modified STAIR criteria:

First we characterized a post-ischemic induction of TTM with regard to time to treatment (≤2 hours; 2< t ≤6 h; >6 h), depth (<29.0°C; 29.1–34.0°C; 34.1–35.6°C) and duration (≤8 hours; 8< t ≤16 h; >16 h). The least temperature reduction from normothermia that we recorded was regarded as the upper limit of depth (reference 180 in list below). Each study was checked against the items below and at the end of the project the scope of testing was summarized across all post-ischemic studies.

Laboratory setting – we recorded whether the regimen was tested in two or more laboratories (by different investigators and in separate laboratories). To be checked, the specific combination of time to treatment, depth and duration, had to be evaluated in two or more laboratories;

Animal species – we recorded whether the regimen was tested in both lissencephalic (rodents, animals with smooth cortex) and gyrencephalic (animals with convoluted cortex) species. To be checked, we required the specific combination to have been tested with both lissencephalic and gyrencephalic animals;

Health of animals – we recorded whether the regimen was tested in comorbid animals (hyperglycemic, aged, hypertensive). To be checked, we required the specific combination to have been tested at least once in a comorbid animal;

Sex of animals – we recorded whether the regimen was tested in male and female animals. To be checked, we required the specific combination to have been tested in both male and female animals;

Outcome measures – we recorded whether the regimen was evaluated with both histology and neurobehaviour. If an outcome was reported with the median and interquartile ranges it was still checked as performed in the STAIR analysis. To be checked, the specific combination had to evaluated with histology and neurobehaviour at least once respectively;

Long-term effect – we recorded whether the regimen was evaluated with a long-term outcome (either histology, mortality or neurobehaviour, defined as 4 weeks or more after ischemia). To be checked, the specific combination had to have an outcome evaluation after 4 weeks at least once;

Route of delivery – we recorded whether the regimen was tested with two or more methods of temperature reduction (e.g. intravascular cooling, surface cooling etc.). To be checked, the specific combination had to be tested in two or more methods;

Global ischemia model – we recorded whether the regimen was tested in two or more models of global ischemia where at least one model of global ischemia is accomplished by induced cardiac arrest. To be checked, we required the specific combination to be tested at least once in a model of selective arrest of cerebral circulation and induced cardiac arrest respectively.

**Full electronic searches**

PubMed: paste entire string in search field.

*(cardiac arrest OR circulatory arrest OR ischemia OR ischaemia OR hypoxia OR anoxia OR infarct OR infarction OR asystole OR resuscitation)* ***AND (****hypothermia[Title/Abstract] OR hyperthermia[Title/Abstract] OR normothermia[Title/Abstract] OR temperature[Title/Abstract] OR thermoregulatory[Title/Abstract] OR thermoregulation[Title/Abstract] OR chill therapy[Title/Abstract] OR cooling[Title/Abstract] OR cryotherapy[Title/Abstract])* ***AND*** *(brain OR hippocampus OR thalamus OR striatum OR cortex OR neuroprotecti* OR cerebral OR cerebrum OR neuron OR neuronal)*

Embase: paste entire string in advanced search field and select “search as broadly as possible” under “mapping”.

*(’cardiac arrest’ OR ’circulatory arrest’ OR ischemia OR ischaemia OR hypoxia OR anoxia OR infarct OR infarction OR asystole OR resuscitation)* ***AND*** *(’hypothermia’:ab,ti OR ’hyperthermia’:ab,ti OR ’normothermia’:ab,ti OR ’temperature’:ab,ti OR ’thermoregulatory’:ab,ti OR ’thermoregulation’:ab,ti OR ’chill therapy’:ab,ti OR ’cooling’:ab,ti OR ’cryotherapy’:ab,ti)* ***AND*** *(brain OR hippocampus OR thalamus OR striatum OR cortex OR neuroprotecti* OR cerebral OR cerebrum OR neuron OR neuronal)*

**Reference-list of all included studies**

1. Abella BS, Zhao D, Alvarado J, Hamann K, Vanden Hoek TL, Becker LB. Intra-arrest cooling improves outcomes in a murine cardiac arrest model. Circulation. 2004;109(22):2786-91.

2. Adachi N, Liu K, Motoki A, Hiraga N, Irisawa Y, Semba K, et al. A comparison of protective effects between L-histidine and hypothermia against ischemia-induced neuronal damage in gerbil hippocampus. Eur J Pharmacol. 2006;546(1-3):69-73.

3. Ao H, Tanimoto H, Yoshitake A, Moon JK, Terasaki H. Long-term mild hypothermia with extracorporeal lung and heart assist improves survival from prolonged cardiac arrest in dogs. Resuscitation. 2001;48(2):163-74.

4. Asai S, Zhao H, Yamashita A, Jike T, Kunimatsu T, Nagata T, et al. Nicergoline enhances glutamate re-uptake and protects against brain damage in rat global brain ischemia. Eur J Pharmacol. 1999;383(3):267-74.

5. Baker AJ, Zornow MH, Grafe MR, Scheller MS, Skilling SR, Smullin DH, et al. Hypothermia prevents ischemia-induced increases in hippocampal glycine concentrations in rabbits. Stroke. 1991;22(5):666-73.

6. Bart RD, Takaoka S, Pearlstein RD, Dexter F, Warner DS. Interactions between hypothermia and the latency to ischemic depolarization: implications for neuroprotection. Anesthesiology. 1998;88(5):1266-73.

7. Beiser DG, Wojcik KR, Zhao D, Orbelyan GA, Hamann KJ, Vanden Hoek TL. Akt1 genetic deficiency limits hypothermia cardioprotection following murine cardiac arrest. Am J Physiol Heart Circ Physiol. 2010;298(6):H1761-8.

8. Boller M, Jung SK, Odegaard S, Muehlmatt A, Katz JM, Becker LB. A combination of metabolic strategies plus cardiopulmonary bypass improves short-term resuscitation from prolonged lethal cardiac arrest. Resuscitation. 2011;82 Suppl 2:S27-34.

9. Boris-Moller F, Smith ML, Siesjo BK. Effects of hypothermia on ischemic brain damage: A comparison between preischemic and postischemic cooling. Neuroscience Research Communications. 1989;5(2):87-94.

10. Boris-Möller F, Kamme F, Wieloch T. The effect of hypothermia on the expression of neurotrophin mRNA in the hippocampus following transient cerebral ischemia in the rat. Molecular Brain Research. 1998;63(1):163-73.

11. Brader EW, Jehle D, Mineo M, Safar P. Protective head-cooling during cardiac arrest and cardiopulmonary resuscitation: the original animal studies. Neurol Int. 2010;2(1):e3.

12. Brient BW, Zimmerman JM, King TC. The effect of prolonged hypothermia and mannitol infusion upon the neurologic sequelae of total cerebral ischemia. Surg Forum. 1965;16:407-8.

13. Buchan A, Pulsinelli WA. Hypothermia but not the N-methyl-D-aspartate antagonist, MK-801, attenuates neuronal damage in gerbils subjected to transient global ischemia. J Neurosci. 1990;10(1):311-6.

14. Busto R, Dietrich WD, Globus MY, Valdes I, Scheinberg P, Ginsberg MD. Small differences in intraischemic brain temperature critically determine the extent of ischemic neuronal injury. J Cereb Blood Flow Metab. 1987;7(6):729-38.

15. Busto R, Dietrich WD, Globus MYT, Ginsberg MD. Postischemic moderate hypothermia inhibits CA1 hippocampal ischemic neuronal injury. Neuroscience Letters. 1989;101(3):299-304.

16. Che D, Li L, Kopil CM, Liu Z, Guo W, Neumar RW, et al. Impact of therapeutic hypothermia onset and duration on survival, neurologic function, and neurodegeneration after cardiac arrest. Crit Care Med. 2011;39(6):1423-30. *Merged with:*

Paine MG, Che D, Li L, Neumar R W. Cerebellar Purkinje cell neurodegeneration after cardiac arrest - effect of therapeutic hypothermia. Resuscitation 83(12): 1511-1516.

17. Chen B, Song FQ, Sun LL, Lei LY, Gan WN, Chen MH, et al. Improved early postresuscitation EEG activity for animals treated with hypothermia predicted 96 hr neurological outcome and survival in a rat model of cardiac arrest. Biomed Res Int. 2013;2013:312137.

18. Chen H, Chopp M, Jiang Q, Garcia JH. Neuronal damage, glial response and cerebral metabolism after hypothermic forebrain ischemia in the rat. Acta Neuropathol. 1992;84(2):184-9.

19. Chenoune M, Lidouren F, Adam C, Pons S, Darbera L, Bruneval P, et al. Ultrafast and whole-body cooling with total liquid ventilation induces favorable neurological and cardiac outcomes after cardiac arrest in rabbits. Circulation. 2011;124(8):901-11, 1-7.

20. Chopp M, Chen H, Dereski MO, Garcia JH. Mild hypothermic intervention after graded ischemic stress in rats. Stroke. 1991;22(1):37-43.

21. Chun-Lin H, Jie W, Xiao-Xing L, Xing L, Yu-Jie L, Hong Z, et al. Effects of therapeutic hypothermia on coagulopathy and microcirculation after cardiopulmonary resuscitation in rabbits. Am J Emerg Med. 2011;29(9):1103-10.

22. Chung SP, Song FQ, Yu T, Weng Y, Sun S, Weil MH, et al. Effect of therapeutic hypothermia vs delta-opioid receptor agonist on post resuscitation myocardial function in a rat model of CPR. Resuscitation. 2011;82(3):350-4.

23. Churn SB, Taft WC, Billingsley MS, Blair RE, DeLorenzo RJ. Temperature modulation of ischemic neuronal death and inhibition of calcium/calmodulin-dependent protein kinase II in gerbils. Stroke. 1990;21(12):1715-21.

24. Clifton GL, Taft WC, Blair RE, Choi SC, DeLorenzo RJ. Conditions for pharmacologic evaluation in the gerbil model of forebrain ischemia. Stroke. 1989;20(11):1545-52.

25. Coimbra C, Drake M, Boris-Moller F, Wieloch T. Long-lasting neuroprotective effect of postischemic hypothermia and treatment with an anti-inflammatory/antipyretic drug. Evidence for chronic encephalopathic processes following ischemia. Stroke. 1996;27(9):1578-85.

26. Coimbra C, Wieloch T. Hypothermia ameliorates neuronal survival when induced 2 hours after ischaemia in the rat. Acta Physiol Scand. 1992;146(4):543-4.

27. Coimbra C, Wieloch T. Moderate hypothermia mitigates neuronal damage in the rat brain when initiated several hours following transient cerebral ischemia. Acta Neuropathol. 1994;87(4):325-31.

28. Coimbra CG, Cavalheiro EA. Protective effect of short-term post-ischemic hypothermia on the gerbil brain. Braz J Med Biol Res. 1990;23(6-7):605-11.

29. Colbourne F, Auer RN, Sutherland GR. Behavioral testing does not exacerbate ischemic CA1 damage in gerbils. Stroke. 1998;29(9):1967-70; discussion 71. *Merged with:*

Colbourne F, Auer RN, Sutherland GR. Characterization of postischemic behavioral deficits in gerbils with and without hypothermic neuroprotection. Brain Res 803(1-2): 69-78.

30. Colbourne F, Corbett D. Delayed and prolonged post-ischemic hypothermia is neuroprotective in the gerbil. Brain Res. 1994;654(2):265-72.

31. Colbourne F, Corbett D. Delayed postischemic hypothermia: a six month survival study using behavioral and histological assessments of neuroprotection. J Neurosci. 1995;15(11):7250-60.

32. Colbourne F, Grooms SY, Zukin RS, Buchan AM, Bennett MV. Hypothermia rescues hippocampal CA1 neurons and attenuates down-regulation of the AMPA receptor GluR2 subunit after forebrain ischemia. Proc Natl Acad Sci U S A. 2003;100(5):2906-10.

33. Colbourne F, Li H, Buchan AM. Indefatigable CA1 sector neuroprotection with mild hypothermia induced 6 hours after severe forebrain ischemia in rats. J Cereb Blood Flow Metab. 1999;19(7):742-9.

34. Colbourne F, Sutherland GR, Auer RN. Electron microscopic evidence against apoptosis as the mechanism of neuronal death in global ischemia. J Neurosci. 1999;19(11):4200-10.

35. Corbett D, Evans S, Thomas C, Wang D, Jonas RA. MK-801 reduced cerebral ischemic injury by inducing hypothermia. Brain Research. 1990;514(2):300-4.

36. Corbett D, Larsen J, Langdon KD. Diazepam delays the death of hippocampal CA1 neurons following global ischemia. Exp Neurol. 2008;214(2):309-14.

37. Corbett D, Nurse S, Colbourne F. Hypothermic neuroprotection. A global ischemia study using 18- to 20-month-old gerbils. Stroke. 1997;28(11):2238-42; discussion 43.

38. Cour M, Jahandiez V, Loufouat J, Ovize M, Argaud L. Minor Changes in Core Temperature Prior to Cardiac Arrest Influence Outcomes: An Experimental Study. Journal of Cardiovascular Pharmacology and Therapeutics. 2014;20(4):407-13.

39. D'Cruz BJ, Logue ES, Falke E, DeFranco DB, Callaway CW. Hypothermia and ERK activation after cardiac arrest. Brain Res. 2005;1064(1-2):108-18.

40. Dai HB, Ji X, Zhu SH, Hu YM, Zhang LD, Miao XL, et al. Hydrogen sulphide and mild hypothermia activate the CREB signaling pathway and prevent ischemia-reperfusion injury. BMC Anesthesiol. 2015;15(1):119.

41. Dai HB, Xu MM, Lv J, Ji XJ, Zhu SH, Ma RM, et al. Mild Hypothermia Combined with Hydrogen Sulfide Treatment During Resuscitation Reduces Hippocampal Neuron Apoptosis Via NR2A, NR2B, and PI3K-Akt Signaling in a Rat Model of Cerebral Ischemia-Reperfusion Injury. Mol Neurobiol. 2016;53(7):4865-73.

42. Darbera L, Chenoune M, Lidouren F, Kohlhauer M, Adam C, Bruneval P, et al. Hypothermic liquid ventilation prevents early hemodynamic dysfunction and cardiovascular mortality after coronary artery occlusion complicated by cardiac arrest in rabbits. Crit Care Med. 2013;41(12):e457-65.

43. Davies LM, MacLellan CL, Corbett DR, Colbourne F. Post-ischemic diazepam does not reduce hippocampal CA1 injury and does not improve hypothermic neuroprotection after forebrain ischemia in gerbils. Brain Res. 2004;1013(2):223-9.

44. De Bow SB, Colbourne F. Delayed transient ischemic attacks kill some CA1 neurons previously salvaged with postischemic hypothermia: neuroprotection undone. Brain Res. 2003;959(1):50-7.

45. Deng R, Koenig MA, Young LM, Jia X. Early Quantitative Gamma-Band EEG Marker is Associated with Outcomes After Cardiac Arrest and Targeted Temperature Management. Neurocrit Care. 2015.

46. Dhruva AJ, Javeri PM, Parulkar GB, Bhatt MM, Sen PK. Fluothane as an anaesthetic adjuvant for prevention of hypoxic brain damage. (An experimental study). J Exp Med Sci. 1961;5:1-7.

47. Dietrich WD, Busto R, Alonso O, Globus MY, Ginsberg MD. Intraischemic but not postischemic brain hypothermia protects chronically following global forebrain ischemia in rats. J Cereb Blood Flow Metab. 1993;13(4):541-9.

48. Dietrich WD, Busto R, Bethea JR. Postischemic hypothermia and IL-10 treatment provide long-lasting neuroprotection of CA1 hippocampus following transient global ischemia in rats. Exp Neurol. 1999;158(2):444-50.

49. Eguchi Y, Yamashita K, Iwamoto T, Ito H. Effects of brain temperature on calmodulin and microtubule-associated protein 2 immunoreactivity in the gerbil hippocampus following transient forebrain ischemia. J Neurotrauma. 1997;14(2):109-18.

50. Eigyo M, Katsuura G, Shintaku H, Shinohara S, Katoh A, Shiomi T, et al. Systemic administration of a cholecystokinin analogue, ceruletide, protects against ischemia-induced neurodegeneration in gerbils. Eur J Pharmacol. 1992;214(2-3):149-58.

51. Gong P, Zhao S, Wang J, Yang Z, Qian J, Wu X, et al. Mild hypothermia preserves cerebral cortex microcirculation after resuscitation in a rat model of cardiac arrest. Resuscitation. 2015;97:109-14.

52. Green EJ, Dietrich WD, van Dijk F, Busto R, Markgraf CG, McCabe PM, et al. Protective effects of brain hypothermia on behavior and histopathology following global cerebral ischemia in rats. Brain Res. 1992;580(1-2):197-204.

53. Green EJ, Pazos AJ, Dietrich WD, McCabe PM, Schneiderman N, Lin B, et al. Combined postischemic hypothermia and delayed MK-801 treatment attenuates neurobehavioral deficits associated with transient global ischemia in rats. Brain Res. 1995;702(1-2):145-52. *Merged with:*

Dietrich WD, Lin B, Globus MYT, Green EJ, Ginsberg MD, Busto R. Effect of delayed MK-801 (dizocilpine) treatment with or without immediate postischemic hypothermia on chronic neuronal survival after global forebrain ischemia in rats. J Cereb Blood Flow Metab 15(6): 960-968.

54. Hachimi-Idrissi S, Van Hemelrijck A, Michotte A, Smolders I, Sarre S, Ebinger G, et al. Postischemic mild hypothermia reduces neurotransmitter release and astroglial cell proliferation during reperfusion after asphyxial cardiac arrest in rats. Brain Res. 2004;1019(1-2):217-25.

55. Hagioka S, Takeda Y, Takata K, Morita K. Nasopharyngeal cooling selectively and rapidly decreases brain temperature and attenuates neuronal damage, even if initiated at the onset of cardiopulmonary resuscitation in rats. Crit Care Med. 2003;31(10):2502-8.

56. Hall ED, Andrus PK, Pazara KE. Protective efficacy of a hypothermic pharmacological agent in gerbil forebrain ischemia. Stroke. 1993;24(5):711-5.

57. Han F, Boller M, Guo W, Merchant RM, Lampe JW, Smith TM, et al. A rodent model of emergency cardiopulmonary bypass resuscitation with different temperatures after asphyxial cardiac arrest. Resuscitation. 2010;81(1):93-9.

58. Hayashida K, Sano M, Kamimura N, Yokota T, Suzuki M, Maekawa Y, et al. H(2) gas improves functional outcome after cardiac arrest to an extent comparable to therapeutic hypothermia in a rat model. J Am Heart Assoc. 2012;1(5):e003459.

59. Hayashida K, Sano M, Kamimura N, Yokota T, Suzuki M, Ohta S, et al. Hydrogen inhalation during normoxic resuscitation improves neurological outcome in a rat model of cardiac arrest independently of targeted temperature management. Circulation. 2014;130(24):2173-80.

60. Hickey RW, Ferimer H, Alexander HL, Garman RH, Callaway CW, Hicks S, et al. Delayed, spontaneous hypothermia reduces neuronal damage after asphyxial cardiac arrest in rats. Crit Care Med. 2000;28(10):3511-6.

61. Hicks SD, DeFranco DB, Callaway CW. Hypothermia during reperfusion after asphyxial cardiac arrest improves functional recovery and selectively alters stress-induced protein expression. J Cereb Blood Flow Metab. 2000;20(3):520-30.

62. Hockings PD, Middleton DA, Patel S, Samson NA, Reid DG, Rose SE, et al. Correlation between high-field T2-weighted MR imaging and histology of ischemic lesions in gerbil brain. Journal of magnetic resonance imaging : JMRI. 1995;5(4):437-42.

63. Horiguchi T, Shimizu K, Ogino M, Yamaguchi N, Suga S, Inamasu J, et al. Neuroprotection role of adenosine under hypothermia in the rat global ischemia involves inhibition of not dopamine release but delayed postischemic hypoperfusion. Brain Res. 2002;952(2):222-31.

64. Hsu CY, Huang CH, Chang WT, Chen HW, Cheng HJ, Tsai MS, et al. Cardioprotective effect of therapeutic hypothermia for postresuscitation myocardial dysfunction. Shock. 2009;32(2):210-6.

65. Hu WW, Du Y, Li C, Song YJ, Zhang GY. Neuroprotection of hypothermia against neuronal death in rat hippocampus through inhibiting the increased assembly of GluR6-PSD95-MLK3 signaling module induced by cerebral ischemia/reperfusion. Hippocampus. 2008;18(4):386-97.

66. Hua R, Li C, Gong P, Tang Z, Mei X, Zhao H. Cerebrospinal fluid biochemistry reflects effects of therapeutic hypothermia after cardiac arrest in a porcine model. American Journal of Emergency Medicine. 2012;30(8):1420-8.

67. Huang CH, Tsai MS, Chiang CY, Su YJ, Wang TD, Chang WT, et al. Activation of mitochondrial STAT-3 and reduced mitochondria damage during hypothermia treatment for post-cardiac arrest myocardial dysfunction. Basic Res Cardiol. 2015;110(6):59.

68. Huang K, Wang Z, Gu Y, Hu Y, Ji Z, Wang S, et al. Glibenclamide Is Comparable to Target Temperature Management in Improving Survival and Neurological Outcome After Asphyxial Cardiac Arrest in Rats. J Am Heart Assoc. 2016;5(7).

69. Iqbal S, Baziany A, Gordon S, Wright S, Hussain M, Miyashita H, et al. Neuroprotective effect of tiagabine in transient forebrain global ischemia: an in vivo microdialysis, behavioral, and histological study. Brain Res. 2002;946(2):162-70.

70. Iwai T, Niwa M, Nakashima M, Kambara T, Yamada H, Tsurumi K, et al. Effect of opioids on delayed neuronal death in the gerbil hippocampus. Life Sci. 1992;50(26):PL239-44.

71. Iwai T, Niwa M, Yamada H, Nozaki M, Tsurumi K. Hypothermic prevention of the hippocampal damage following ischemia in Mongolian gerbils comparison between intraischemic and brief postischemic hypothermia. Life Sci. 1993;52(12):1031-8.

72. Janata A, Drabek T, Magnet IA, Stezoski JP, Janesko-Feldman K, Popp E, et al. Extracorporeal versus conventional cardiopulmonary resuscitation after ventricular fibrillation cardiac arrest in rats: a feasibility trial. Crit Care Med. 2013;41(9):e211-22.

73. Jensen LH, Moller A. Time- and temperature-dependent forebrain ischemic damage in Mongolian gerbils. Acta Neurol Scand. 1992;85(3):187-90.

74. Jia X, Koenig MA, Nickl R, Zhen G, Thakor NV, Geocadin RG. Early electrophysiologic markers predict functional outcome associated with temperature manipulation after cardiac arrest in rats. Crit Care Med. 2008;36(6):1909-16.

75. Jia X, Koenig MA, Shin HC, Zhen G, Yamashita S, Thakor NV, et al. Quantitative EEG and neurological recovery with therapeutic hypothermia after asphyxial cardiac arrest in rats. Brain Res. 2006;1111(1):166-75.

76. Jia X, Koenig MA, Venkatraman A, Thakor NV, Geocadin RG. Post-cardiac arrest temperature manipulation alters early EEG bursting in rats. Resuscitation. 2008;78(3):367-73.

77. Kang X, Jia X, Geocadin RG, Thakor NV, Maybhate A. Multiscale entropy analysis of EEG for assessment of post-cardiac arrest neurological recovery under hypothermia in rats. IEEE Trans Biomed Eng. 2009;56(4):1023-31.

78. Katz LM, Frank JE, Glickman LT, Jr GM, Lambert BH, Gordon CJ. Effect of a pharmacologically induced decrease in core temperature in rats resuscitated from cardiac arrest. Resuscitation. 2015;92:26-31.

79. Katz LM, Young A, Frank JE, Wang Y, Park K. Neurotensin-induced hypothermia improves neurologic outcome after hypoxic-ischemia. Crit Care Med. 2004;32(3):806-10.

80. Kawai K, Nakayama H, Tamura A. Limited but significant protective effect of hypothermia on ultra-early-type ischemic neuronal injury in the thalamus. J Cereb Blood Flow Metab. 1997;17(5):543-52.

81. Kelly S, Cheng D, Steinberg GK, Yenari MA. Mild hypothermia decreases GSK3beta expression following global cerebral ischemia. Neurocrit Care. 2005;2(2):212-7.

82. Kida K, Shirozu K, Yu B, Mandeville JB, Bloch KD, Ichinose F. Beneficial effects of nitric oxide on outcomes after cardiac arrest and cardiopulmonary resuscitation in hypothermia-treated mice. Anesthesiology. 2014;120(4):880-9.

83. Kim JY, Kim N, Yenari MA, Chang W. Mild Hypothermia Suppresses Calcium-Sensing Receptor (CaSR) Induction Following Forebrain Ischemia While Increasing GABA-B Receptor 1 (GABA-B-R1) Expression. Transl Stroke Res. 2011;2(2):195-201.

84. Kim T, Paine MG, Meng H, Xiaodan R, Cohen J, Jinka T, et al. Combined intra- and post-cardiac arrest hypothermic-targeted temperature management in a rat model of asphyxial cardiac arrest improves survival and neurologic outcome compared to either strategy alone. Resuscitation. 2016;107:94-101.

85. Kimura T, Sako K, Tanaka K, Kusakabe M, Tanaka T, Nakada T. Effect of mild hypothermia on energy state recovery following transient forebrain ischemia in the gerbil. Exp Brain Res. 2002;145(1):83-90.

86. Kohlhauer M, Lidouren F, Remy-Jouet I, Mongardon N, Adam C, Bruneval P, et al. Hypothermic Total Liquid Ventilation Is Highly Protective Through Cerebral Hemodynamic Preservation and Sepsis-Like Mitigation After Asphyxial Cardiac Arrest. Crit Care Med. 2015.

87. Krieglstein J, Seif El Nasr M, Lippert K. Neuroprotection by memantine as increased by hypothermia and nimodipine. European Journal of Pharmaceutical Sciences. 1997;5(2):71-7.

88. Kuboyama K, Safar P, Oku K, Obrist W, Leonov Y, Sterz F, et al. Mild hypothermia after cardiac arrest in dogs does not affect postarrest cerebral oxygen uptake/delivery mismatching. Resuscitation. 1994;27(3):231-44.

89. Kuboyama K, Safar P, Radovsky A, Tisherman SA, Stezoski SW, Alexander H. Delay in cooling negates the beneficial effect of mild resuscitative cerebral hypothermia after cardiac arrest in dogs: a prospective, randomized study. Crit Care Med. 1993;21(9):1348-58.

90. Kuluz JW, Gregory GA, Yu AC, Chang Y. Selective brain cooling during and after prolonged global ischemia reduces cortical damage in rats. Stroke. 1992;23(12):1792-6; discussion 7.

91. Kunimatsu T, Yamashita A, Kitahama H, Misaki T, Yamamoto T. Measurement of cerebral reactive hyperemia at the initial post-ischemia reperfusion stage under normothermia and moderate hypothermia in rats. J Oral Sci. 2009;51(4):615-21.

92. Kwon JY, Bacher A, Deyo DJ, Disterhoft JF, Uchida T, Zornow MH. Effects of pentobarbital and isoflurane on conditioned learning after transient global cerebral ischemia in rabbits. Anesthesiology. 2000;92(1):171-7.

93. Kwon JY, Bacher A, Deyo DJ, Grafe MR, Disterhoft JF, Uchida T, et al. Effects of hypothermia and lamotrigine on trace-conditioned learning after global cerebral ischemia in rabbits. Exp Neurol. 1999;159(1):105-13.

94. Lagina AT, 3rd, Calo L, Deogracias M, Sanderson T, Kumar R, Wider J, et al. Combination therapy with insulin-like growth factor-1 and hypothermia synergistically improves outcome after transient global brain ischemia in the rat. Acad Emerg Med. 2013;20(4):344-51.

95. Lagina AT, Deogracias M, Reed K, Bazzi D, Chepuri R, Foster L, et al. The "Refrige-a-RAT-or": an accurate, inexpensive, and clinically relevant small animal model of therapeutic hypothermia. Acad Emerg Med. 2012;19(4):402-8.

96. Lasarzik I, Winkelheide U, Thal SC, Benz N, Lorscher M, Jahn-Eimermacher A, et al. Mild hypothermia has no long-term impact on postischemic neurogenesis in rats. Anesth Analg. 2009;109(5):1632-9.

97. Lee JH, Kim K, Jo YH, Lee MJ, Hwang JE, Kim MA. Effect of valproic acid combined with therapeutic hypothermia on neurologic outcome in asphyxial cardiac arrest model of rats. Am J Emerg Med. 2015;33(12):1773-9.

98. Lei B, Adachi N, Arai T. The effect of hypothermia on H2O2 production during ischemia and reperfusion: a microdialysis study in the gerbil hippocampus. Neurosci Lett. 1997;222(2):91-4.

99. Leonov Y, Sterz F, Safar P, Radovsky A. Moderate hypothermia after cardiac arrest of 17 minutes in dogs. Effect on cerebral and cardiac outcome. Stroke. 1990;21(11):1600-6.

100. Leonov Y, Sterz F, Safar P, Radovsky A, Oku K, Tisherman S, et al. Mild cerebral hypothermia during and after cardiac arrest improves neurologic outcome in dogs. J Cereb Blood Flow Metab. 1990;10(1):57-70.

101. Li LX, Campbell K, Zhao S, Knuckey NW, Meloni BP. Comparison of the efficacy of mild hypothermia (35 degrees c) and moderate hypothermia (33 degrees c), alone or combined with magnesium treatment, when commenced 2 or 4 hours after global cerebral ischemia in rats. Ther Hypothermia Temp Manag. 2011;1(3):151-8.

102. Li PA, He QP, Miyashita H, Howllet W, Siesjo BK, Shuaib A. Hypothermia ameliorates ischemic brain damage and suppresses the release of extracellular amino acids in both normo- and hyperglycemic subjects. Exp Neurol. 1999;158(1):242-53.

103. Li PA, He QP, Siesjo BK. Effects of intracarotid arterial injection of cyclosporin A and spontaneous hypothermia on brain damage incurred after a long period of global ischemia. Brain Res. 2001;890(2):306-13.

104. Liu X, Wang M, Chen H, Guo Y, Ma F, Shi F, et al. Hypothermia protects the brain from transient global ischemia/reperfusion by attenuating endoplasmic reticulum response-induced apoptosis through CHOP. PLoS One. 2013;8(1):e53431.

105. Logue ES, McMichael MJ, Callaway CW. Comparison of the effects of hypothermia at 33 degrees C or 35 degrees C after cardiac arrest in rats. Acad Emerg Med. 2007;14(4):293-300.

106. Lu J, Qian HY, Liu LJ, Zhou BC, Xiao Y, Mao JN, et al. Mild hypothermia alleviates excessive autophagy and mitophagy in a rat model of asphyxial cardiac arrest. Neurol Sci. 2014;35(11):1691-9. *Merged with:*

Lu J, Shen Y, Qian HY, Liu LJ, Zhou BC, Xiao Y, et al. Effects of mild hypothermia on the ROS and expression caspase-3 mRNA and LC3 of hippocampus nerve cells in rats after cardiopulmonary resuscitation. World J Emerg Med 5(4): 298-305.

107. Lu X, Ma L, Sun S, Xu J, Zhu C, Tang W. The effects of the rate of postresuscitation rewarming following hypothermia on outcomes of cardiopulmonary resuscitation in a rat model. Crit Care Med. 2014;42(2):e106-13.

108. Lundgren J, Smith ML, Siesjo BK. Influence of moderate hypothermia on ischemic brain damage incurred under hyperglycemic conditions. Exp Brain Res. 1991;84(1):91-101.

109. Martinez NS, Machado JM, Perez-Saad H, Coro-Antich RM, Berlanga-Acosta JA, Salgueiro SR, et al. Global brain ischemia in Mongolian gerbils: assessing the level of anastomosis in the cerebral circle of Willis. Acta Neurobiol Exp (Wars). 2012;72(4):377-84.

110. Matsuda T. Effects of hypothermia on c-fos and zif/268 gene expression following rat forebrain ischemia. J Anesth. 1999;13(2):99-106.

111. Minamisawa H, Nordstrom CH, Smith ML, Siesjo BK. The influence of mild body and brain hypothermia on ischemic brain damage. J Cereb Blood Flow Metab. 1990;10(3):365-74.

112. Minamisawa H, Smith ML, Siesjo BK. The effect of mild hyperthermia and hypothermia on brain damage following 5, 10, and 15 minutes of forebrain ischemia. Ann Neurol. 1990;28(1):26-33.

113. Mitani A, Kataoka K. Critical levels of extracellular glutamate mediating gerbil hippocampal delayed neuronal death during hypothermia: brain microdialysis study. Neuroscience. 1991;42(3):661-70.

114. Miyamoto O, Nakamura T, Yamagami S, Negi T, Tokuda M, Matsui H, et al. Depression of long term potentiation in gerbil hippocampus following postischemic hypothermia. Brain Res. 2000;873(1):168-72.

115. Miyazawa T, Bonnekoh P, Widmann R, Hossmann KA. Heating of the brain to maintain normothermia during ischemia aggravates brain injury in the rat. Acta Neuropathol. 1993;85(5):488-94.

116. Nakajima Y, Fujimiya M, Maeda T, Mori A. Morphological investigation of the neuroprotective effects of graded hypothermia after diverse periods of global cerebral ischemia in gerbils. Brain Res. 1997;765(1):113-21.

117. Nakamura T, Miyamoto O, Kawai N, Negi T, Itano T, Nagao S. Long-term activation of the glutamatergic system associated with N-methyl-D-aspartate receptors after postischemic hypothermia in gerbils. Neurosurgery. 2001;49(3):706-13; discussion 13-4.

118. Nakamura T, Miyamoto O, Yamagami S, Hayashida Y, Itano T, Nagao S. Influence of rewarming conditions after hypothermia in gerbils with transient forebrain ischemia. J Neurosurg. 1999;91(1):114-20.

119. Nakamura T, Miyamoto O, Yamagami S, Toyoshima T, Negi T, Itano T, et al. The chronic cell death with DNA fragmentation after post-ischaemic hypothermia in the gerbil hippocampus. Acta Neurochir (Wien). 1999;141(4):407-12; discussion 12-3.

120. Nakane M, Kubota M, Nakagomi T, Tamura A, Hisaki H, Ueta N. Rewarming eliminates the protective effect of cooling against delayed neuronal death. Neuroreport. 2001;12(11):2439-42.

121. Natale JA, D'Alecy LG. Protection from cerebral ischemia by brain cooling without reduced lactate accumulation in dogs. Stroke. 1989;20(6):770-7.

122. Noguchi K, Matsumoto N, Shiozaki T, Tasaki O, Ogura H, Kuwagata Y, et al. Effects of timing and duration of hypothermia on survival in an experimental gerbil model of global ischaemia. Resuscitation. 2011;82(4):481-6.

123. Nurse S, Corbett D. Direct measurement of brain temperature during and after intraischemic hypothermia: correlation with behavioral, physiological, and histological endpoints. J Neurosci. 1994;14(12):7726-34.

124. Nurse S, Corbett D. Neuroprotection after several days of mild, drug-induced hypothermia. J Cereb Blood Flow Metab. 1996;16(3):474-80.

125. Oh JS, Kim SW, Cho HJ, Kyong YY, Oh YM, Choi SM, et al. Combination treatment with 17beta-estradiol and therapeutic hypothermia for transient global cerebral ischemia in rats. Am J Emerg Med. 2013;31(1):154-60.

126. Olsson T, Wieloch T, Smith ML. Brain damage in a mouse model of global cerebral ischemia. Effect of NMDA receptor blockade. Brain Res. 2003;982(2):260-9.

127. Ooboshi H, Ibayashi S, Takano K, Sadoshima S, Kondo A, Uchimura H, et al. Hypothermia inhibits ischemia-induced efflux of amino acids and neuronal damage in the hippocampus of aged rats. Brain Res. 2000;884(1--2):23-30.

128. Otsuka N, Tsuritani K, Sakurai T, Kato K, Matoba R, Itoh J, et al. Transcriptional induction and translational inhibition of Arc and Cugbp2 in mice hippocampus after transient global ischemia under normothermic condition. Brain Res. 2009;1287:136-45.

129. Pazos AJ, Green EJ, Busto R, McCabe PM, Baena RC, Ginsberg MD, et al. Effects of combined postischemic hypothermia and delayed N-tert-butyl-alpha-pheylnitrone (PBN) administration on histopathologicaland behavioral deficits associated with transient global ischemia in rats. Brain Res. 1999;846(2):186-95.

130. Pellegrini-Giampietro DE, Cozzi A, Moroni F. The glycine antagonist and free radical scavenger 7-Cl-thio-kynurenate reduces CA1 ischemic damage in the gerbil. Neuroscience. 1994;63(3):701-9.

131. Sano T, Drummond JC, Patel PM, Grafe MR, Watson JC, Cole DJ. A comparison of the cerebral protective effects of isoflurane and mild hypothermia in a model of incomplete forebrain ischemia in the rat. Anesthesiology. 1992;76(2):221-8.

132. Sarnowska A, Beresewicz M, Zablocka B, Domanska-Janik K. Diazepam neuroprotection in excitotoxic and oxidative stress involves a mitochondrial mechanism additional to the GABAAR and hypothermic effects. Neurochem Int. 2009;55(1-3):164-73.

133. Satoh K, Niwa M, Goda W, Binh NH, Nakashima M, Takamatsu M, et al. Galectin-3 expression in delayed neuronal death of hippocampal CA1 following transient forebrain ischemia, and its inhibition by hypothermia. Brain Res. 2011;1382:266-74.

134. Schneider A, Teschendorf P, Vogel P, Russ N, Knapp J, Bottiger BW, et al. Facilitation of hypothermia by quinpirole and 8-OH-DPAT in a rat model of cardiac arrest. Resuscitation. 2012;83(2):232-7.

135. Seif el Nasr M, Nuglisch J, Krieglstein J. Prevention of ischemia-induced cerebral hypothermia by controlling the environmental temperature. J Pharmacol Toxicol Methods. 1992;27(1):23-6.

136. Sheng H, Laskowitz DT, Pearlstein RD, Warner DS. Characterization of a recovery global cerebral ischemia model in the mouse. J Neurosci Methods. 1999;88(1):103-9.

137. Shuaib A, Waqar T, Wishart T, Kanthan R. Post-ischemic therapy with CGS-19755 (alone or in combination with hypothermia) in gerbils. Neurosci Lett. 1995;191(1-2):87-90.

138. Siemkowicz E, Haider A. Post-ischemic hypothermia ameliorates ischemic brain damage but not post-ischemic audiogenic seizures in rats. Resuscitation. 1995;30(1):61-7.

139. Silasi G, Colbourne F. Therapeutic hypothermia influences cell genesis and survival in the rat hippocampus following global ischemia. J Cereb Blood Flow Metab. 2011;31(8):1725-35.

140. Silasi G, Colbourne F. Unilateral brain hypothermia as a method to examine efficacy and mechanisms of neuroprotection against global ischemia. Ther Hypothermia Temp Manag. 2011;1(2):87-94.

141. Silasi G, Klahr AC, Hackett MJ, Auriat AM, Nichol H, Colbourne F. Prolonged therapeutic hypothermia does not adversely impact neuroplasticity after global ischemia in rats. J Cereb Blood Flow Metab. 2012;32(8):1525-34.

142. Sterz F, Safar P, Tisherman S, Radovsky A, Kuboyama K, Oku KI. Mild hypothermic cardiopulmonary resuscitation improves outcome after prolonged cardiac arrest in dogs. Critical Care Medicine. 1991;19(3):379-89.

143. Sun S, Tang W, Song F, Yu T, Ristagno G, Shan Y, et al. The effects of epinephrine on outcomes of normothermic and therapeutic hypothermic cardiopulmonary resuscitation. Crit Care Med. 2010;38(11):2175-80.

144. Takasu A, Yagi KI, Okada Y. Effect of mild hypothermia on ischemia-induced release of endothelin-1 in dog brain. Resuscitation. 1996;31(1):59-64.

145. Takata K, Takeda Y, Sato T, Nakatsuka H, Yokoyama M, Morita K. Effects of hypothermia for a short period on histologic outcome and extracellular glutamate concentration during and after cardiac arrest in rats. Crit Care Med. 2005;33(6):1340-5.

146. Takeda Y, Hashimoto H, Fumoto K, Danura T, Naito H, Morimoto N, et al. Effects of pharyngeal cooling on brain temperature in primates and humans: a study for proof of principle. Anesthesiology. 2012;117(1):117-25.

147. Takeda Y, Namba K, Higuchi T, Hagioka S, Takata K, Hirakawa M, et al. Quantitative evaluation of the neuroprotective effects of hypothermia ranging from 34 degrees C to 31 degrees C on brain ischemia in gerbils and determination of the mechanism of neuroprotection. Crit Care Med. 2003;31(1):255-60.

148. Tang ZR, Li CS, Zhao H, Gong P, Zhang MY, Su ZY, et al. Effects of hypothermia on brain injury assessed by magnetic resonance imaging after cardiopulmonary resuscitation in a porcine model of cardiac arrest. Am J Emerg Med. 2013;31(1):86-93.

149. Tasdemiroglu E. Mild hypothermia fails to protect late hippocampal neuronal loss following forebrain cerebral ischaemia in rats. Acta Neurochir (Wien). 1996;138(5):570-8; discussion 8-9.

150. Tsai MS, Huang CH, Tsai CY, Chen HW, Cheng HJ, Hsu CY, et al. Combination of intravenous ascorbic acid administration and hypothermia after resuscitation improves myocardial function and survival in a ventricular fibrillation cardiac arrest model in the rat. Acad Emerg Med. 2014;21(3):257-65.

151. Tsai MS, Huang CH, Yu PH, Tsai CY, Chen HW, Cheng HJ, et al. Prolonged cooling duration mitigates myocardial and cerebral damage in cardiac arrest. Am J Emerg Med. 2015.

152. Tsuchiya D, Hong S, Suh SW, Kayama T, Panter SS, Weinstein PR. Mild hypothermia reduces zinc translocation, neuronal cell death, and mortality after transient global ischemia in mice. J Cereb Blood Flow Metab. 2002;22(10):1231-8.

153. Uno H, Kobayashi H, Handa Y, Kabuto M, Kubota T. Alterations of calcium/calmodulin-dependent protein kinase II activity in ischaemia-induced neuronal death and neuronal protection against ischaemia in the gerbil hippocampus. Acta Neurochir (Wien). 1999;141(3):287-94.

154. Vosler PS, Logue ES, Repine MJ, Callaway CW. Delayed hypothermia preferentially increases expression of brain-derived neurotrophic factor exon III in rat hippocampus after asphyxial cardiac arrest. Brain Res Mol Brain Res. 2005;135(1-2):21-9.

155. Wada K, Nishi D, Kitamura T, Ono K, Takahara T, Shirotani T, et al. Hyperbaric oxygenation therapy enhances the protective effect of moderate hypothermia against forebrain ischemia in the gerbil hippocampus. Undersea Hyperb Med. 2006;33(6):399-405.

156. Wang P, Jia L, Chen B, Zhang L, Liu J, Long J, et al. Hydrogen Inhalation is Superior to Mild Hypothermia in Improving Cardiac Function and Neurological Outcome in an Asphyxial Cardiac Arrest Model of Rats. Shock. 2016;46(3):312-8.

157. Wang P, Zhang L, Gong Y, Zhang H, Wang X, Li Y. Impacts of systemic hypertension and left ventricular hypertrophy on outcome of cardiopulmonary resuscitation and therapeutic hypothermia in a cardiac arrest model of rat. Shock. 2016;45(4):434-40.

158. Warner MA, Neill KH, Nadler JV, Crain BJ. Regionally selective effects of NMDA receptor antagonists against ischemic brain damage in the gerbil. J Cereb Blood Flow Metab. 1991;11(4):600-10. *Merged with:*

Warner MA, Nadler JV, Crain BJ. Effects of NMDA receptor antagonists and body temperature in the gerbil carotid occlusion model of transient forebrain ischemia. Prog Clin Biol Res 361: 409-14.

159. Webster CM, Kelly S, Koike MA, Chock VY, Giffard RG, Yenari MA. Inflammation and NFkappaB activation is decreased by hypothermia following global cerebral ischemia. Neurobiol Dis. 2009;33(2):301-12.

160. Wei G, Dore S. Importance of normothermia control in investigating delayed neuronal injury in a mouse global ischemia model. J Neurosci Methods. 2010;185(2):230-5.

161. Weinrauch V, Safar P, Tisherman S, Kuboyama K, Radovsky A. Beneficial effect of mild hypothermia and detrimental effect of deep hypothermia after cardiac arrest in dogs. Stroke. 1992;23(10):1454-62.

162. Welsh FA, Harris VA. Postischemic hypothermia fails to reduce ischemic injury in gerbil hippocampus. J Cereb Blood Flow Metab. 1991;11(4):617-20.

163. Welsh FA, Sims RE, Harris VA. Mild hypothermia prevents ischemic injury in gerbil hippocampus. J Cereb Blood Flow Metab. 1990;10(4):557-63.

164. Wolfe KB. Effect of hypothermia on cerebral damage resulting from cardiac arrest. Am J Cardiol. 1960;6:809-12.

165. Wu L, Sun HL, Gao Y, Hui KL, Xu MM, Zhong H, et al. Therapeutic Hypothermia Enhances Cold-Inducible RNA-Binding Protein Expression and Inhibits Mitochondrial Apoptosis in a Rat Model of Cardiac Arrest. Mol Neurobiol. 2016.

166. Xiang Z, Thomas S, Pasinetti G. Increased neuronal injury in transgenic mice with neuronal overexpression of human cyclooxygenase-2 is reversed by hypothermia and rofecoxib treatment. Curr Neurovasc Res. 2007;4(4):274-9.

167. Xiao F, Safar P, Radovsky A. Mild protective and resuscitative hypothermia for asphyxial cardiac arrest in rats. Am J Emerg Med. 1998;16(1):17-25.

168. Yamamoto H, Mitani A, Cui Y, Takechi S, Irita J, Suga T, et al. Neuroprotective effect of mild hypothermia cannot be explained in terms of a reduction of glutamate release during ischemia. Neuroscience. 1999;91(2):501-9.

169. Yamashita K, Eguchi Y, Kajiwara K, Ito H. Mild hypothermia ameliorates ubiquitin synthesis and prevents delayed neuronal death in the gerbil hippocampus. Stroke. 1991;22(12):1574-81.

170. Yamashita S, Miyamoto O, Janjua NA, Tomizawa K, Matsui H, Nakamura T, et al. Role of the hippocampal CA2 region following postischemic hypothermia in gerbil. Brain Res Mol Brain Res. 2003;111(1-2):8-16.

171. Ye S, Weng Y, Sun S, Chen W, Wu X, Li Z, et al. Comparison of the durations of mild therapeutic hypothermia on outcome after cardiopulmonary resuscitation in the rat. Circulation. 2012;125(1):123-9.

172. Yli-Hankala A, Edmonds HL, Jr., Jiang YD, Higham HE, Zhang PY. Outcome effects of different protective hypothermia levels during cardiac arrest in rats. Acta Anaesthesiol Scand. 1997;41(4):511-5.

173. Zhang H, Xu G, Zhang J, Murong S, Mei Y, Tong E. Mild hypothermia reduces ischemic neuron death via altering the expression of p53 and bcl-2. Neurol Res. 2010;32(4):384-9.

174. Zhang JC, Lu W, Xie XM, Pan H, Wu ZQ, Yang GT. Mild hypothermia attenuates post-resuscitation brain injury through a V-ATPase mechanism in a rat model of cardiac arrest. Genet Mol Res. 2016;15(2).

175. Zhang Z, Sobel RA, Cheng D, Steinberg GK, Yenari MA. Mild hypothermia increases Bcl-2 protein expression following global cerebral ischemia. Brain Res Mol Brain Res. 2001;95(1-2):75-85.

176. Zhao D, Abella BS, Beiser DG, Alvarado JP, Wang H, Hamann KJ, et al. Intra-arrest cooling with delayed reperfusion yields higher survival than earlier normothermic resuscitation in a mouse model of cardiac arrest. Resuscitation. 2008;77(2):242-9.

177. Zhao H, Chen Y. Effects of mild hypothermia therapy on the levels of glutathione in rabbit blood and cerebrospinal fluid after cardiopulmonary resuscitation. Iran J Basic Med Sci. 2015;18(2):194-8. *Merged with:*

Zhao H, Chen Y, Jin Y. The effect of therapeutic hypothermia after cardiopulmonary resuscitation on ICAM-1 and NSE levels in sudden cardiac arrest rabbits. Int J Neurosci 125(7): 540-546.

178. Zhao H, Li CS, Gong P, Tang ZR, Hua R, Mei X, et al. Molecular mechanisms of therapeutic hypothermia on neurological function in a swine model of cardiopulmonary resuscitation. Resuscitation. 2012;83(7):913-20.

179. Zhu H, Meloni BP, Bojarski C, Knuckey MW, Knuckey NW. Post-ischemic modest hypothermia (35°C) combined with intravenous magnesium is more effective at reducing CA1 neuronal death than either treatment used alone following global cerebral ischemia in rats. Experimental Neurology. 2005;193(2):361-8.

180. Zhu H, Meloni BP, Moore SR, Majda BT, Knuckey NW. Intravenous administration of magnesium is only neuroprotective following transient global ischemia when present with post-ischemic mild hypothermia. Brain Res. 2004;1014(1-2):53-60.

181. Zimmerman JM, Spencer FC. The influence of hypothermia on cerebral injury resulting from circulatory occlusion. Surg Forum. 1958;9:216-8.
